# Association of Higher-Dose Fluoroquinolone Therapy With Serious Adverse Events in Older Adults With Advanced Chronic Kidney Disease

**DOI:** 10.1001/jamanetworkopen.2022.24892

**Published:** 2022-08-02

**Authors:** Flory Tsobo Muanda, Manish M. Sood, Matthew A. Weir, Jessica M. Sontrop, Fatemeh Ahmadi, Elisa Yoo, Richard B. Kim, Michael S. Silverman, Gregory A. Knoll, Amit X. Garg

**Affiliations:** 1 ICES Western, Victoria Hospital, London, Ontario, Canada; 2 Department of Epidemiology and Biostatistics, Western University, London, Ontario, Canada; 3 Division of Nephrology, Department of Medicine, University of Ottawa, Ottawa, Ontario, Canada; 4 Division of Nephrology, Department of Medicine, Schulich School of Medicine & Dentistry, Western University, London, Ontario, Canada; 5 Division of Clinical Pharmacology, Department of Medicine, Schulich School of Medicine & Dentistry, Western University, London, Ontario, Canada.; 6 Division of Infectious Diseases, Department of Medicine, Schulich School of Medicine & Dentistry, Western University, London, Ontario, Canada

## Abstract

**Importance:**

Population-based data are needed to inform the safe prescribing of fluoroquinolone antibiotics to patients with advanced chronic kidney disease (CKD).

**Objective:**

To quantify the 14-day risk of a hospital visit with nervous system and/or psychiatric disorders, hypoglycemia, or a collagen-associated event in patients with advanced CKD newly prescribed a fluoroquinolone at a higher vs a lower dose.

**Design, Setting, and Participants:**

This population-based cohort study in Ontario, Canada (January 1, 2008, to March 17, 2020) used linked health care data to identify new users of fluoroquinolone antibiotics. Participants included adults 66 years or older with advanced CKD (an estimated glomerular filtration rate [eGFR] <30 mL/min/1.73 m^2^ but not receiving dialysis). Data analysis was performed from January 1 to April 30, 2021.

**Exposures:**

A new prescription for a higher-dose fluoroquinolone (ciprofloxacin, 501-1000 mg/d; levofloxacin, 501-750 mg/d; or norfloxacin, 401-800 mg/d) vs a lower-dose fluoroquinolone (ciprofloxacin, 500 mg/d; levofloxacin, 250-500 mg/d; or norfloxacin, 400 mg/d).

**Main Outcomes and Measure:**

The primary outcome was the 14-day risk of a hospital visit with nervous system and/or psychiatric disorders, hypoglycemia, or a collagen-associated event. Secondary outcomes included a hospital visit with sepsis, retinal detachment or other tendinopathies, all-cause hospitalization, all-cause mortality, and sudden cardiac death. Inverse probability of treatment weighting on the propensity score was used to balance comparison groups on baseline health. Weighted risk ratios and risk differences were obtained using modified Poisson regression and binomial regression, respectively.

**Results:**

Of 11 917 patients (median age, 83 years [IQR, 77-89 years]; 7438 women [62.4%]; median eGFR, 25 [IQR, 21-28] mL/min/1.73 m^2^) included in the analysis, 5482 (46.0%) received a higher-dose and 6435 (54.0%) received a lower-dose fluoroquinolone. After weighting, the primary composite outcome—a hospital visit with nervous system and/or psychiatric disorders, hypoglycemia, or a collagen-associated event—occurred in 68 of 5482 patients (1.2%) treated with a higher-dose fluoroquinolone and in 47 of 5516 (0.9%) treated with a lower-dose fluoroquinolone (weighted risk ratio, 1.45 [95% CI, 1.01-2.08]; weighted risk difference, 0.39% [95% CI, 0.01%-0.76%]). The risk of sepsis, retinal detachment, all-cause hospitalization, all-cause mortality, and sudden cardiac death did not differ significantly between groups.

**Conclusions and Relevance:**

These findings suggest that older patients with advanced CKD who were prescribed a fluoroquinolone at a higher-than-recommended dose were significantly more likely to experience the composite outcome of a hospital visit with nervous system and/or psychiatric disorders, hypoglycemia, or a collagen-associated event, although the absolute risk of these events was less than 2%.

## Key Points

**Question:**

Is treatment with fluoroquinolone at a higher vs a lower dose associated with increased risk of serious adverse events among older patients with advanced chronic kidney disease (CKD)?

**Findings:**

In this cohort study of 11 917 older patients with advanced CKD, treatment with fluoroquinolone at a higher vs a lower dose was associated with increased risk of a composite outcome of a hospital visit with nervous system and/or psychiatric disorders, hypoglycemia, or a collagen-associated event.

**Meaning:**

These findings suggest that fluoroquinolones should be prescribed cautiously and at lower doses among older adults with advanced CKD.

## Teaser

This cohort study examines the risks for adverse effects of treatment with high- vs low-dose fluoroquinolone antibiotics among patients 66 years or older with advanced chronic kidney disease (CKD).

## Introduction

Fluoroquinolones are among the most commonly prescribed broad-spectrum antibiotics worldwide.^1,2^ In the US, 6.7 million ciprofloxacin prescriptions and 3.8 million levofloxacin prescriptions were filled in 2018.^3^ Indications for these antibiotics include urinary tract infection, respiratory tract infection, gastrointestinal tract infection, and skin and soft tissue infection.^1,4^ Fluoroquinolone use is associated with a number of rare but serious adverse events including nervous system and/or psychiatric disorders (eg, hospitalization with delirium or peripheral neuropathy),^4,5,6^ hypoglycemia,^4,7^ and collagen-associated adverse events (eg, Achilles tendon rupture, abdominal aortic aneurysm rupture).^4,8,9,10^ These risks have been reported in population-based studies and meta-analyses of randomized clinical trials and observational studies.^4,5,6,7,8,9,11,12,13^ The US Food and Drug Administration (FDA) has issued several black-box warnings about the risks of fluoroquinolone use.^14,15,16,17,18,19^ Given the potential for harm, fluoroquinolones are typically reserved to treat more severe bacterial infections in which the benefits clearly outweigh the risks.^4^

Except for moxifloxacin, fluoroquinolones are primarily eliminated by the kidney, and excretion is therefore slower in patients with reduced kidney function.^20,21^ The product monograph and prescribing guidelines recommend that patients with reduced kidney function take fluoroquinolones at lower doses, particularly patients with an estimated glomerular filtration rate (eGFR) of less than 30 mL/min per 1.73 m^2^ (eTable 1 in the Supplement). However, in practice, fluoroquinolones are frequently prescribed to these patients at higher than recommended doses.^22^ Adverse events associated with fluoroquinolone use in patients with chronic kidney disease (CKD) have been reported in 30 case reports and in 1 cohort study of 264 968 patients receiving hemodialysis (a literature search and summary of studies are provided in eTables 2 and 3, respectively, in the Supplement).^23^

To inform safe fluoroquinolone prescribing in patients with advanced CKD, we conducted a population-based study of older adults with an eGFR of less than 30 mL/min per 1.73 m^2^ (excluding those receiving dialysis) and examined the risk of serious adverse events in patients who were treated with a fluoroquinolone at a higher vs a lower dose. We defined a higher-dose fluoroquinolone as ciprofloxacin, 501 to 1000 mg/d; levofloxacin, 501 to 750 mg/d; or norfloxacin, 401 to 800 mg/d. We defined a lower-dose fluoroquinolone as ciprofloxacin, 500 mg/d; levofloxacin, 250 to 500 mg/d; or norfloxacin, 400 mg/d. The primary outcome was the 14-day risk of a hospital visit with nervous system and/or psychiatric disorders, hypoglycemia, or collagen-associated events.

## Methods

### Study Design and Setting

We conducted a new-user, population-based cohort study using linked administrative health care databases in the province of Ontario, Canada, from January 1, 2008, to March 17, 2020. All Ontario residents (approximately 15 million) have universal access to hospital care and physician services through a government-funded single-payer system.^24^ Patients aged 65 years and older (approximately 2.2 million) also receive universal prescription drug coverage. The use of data in this study was authorized under section 45 of Ontario’s Personal Health Information Protection Act, which does not require review by a research ethics board or informed consent. This study followed the Strengthening the Reporting of Observational Studies in Epidemiology (STROBE) reporting guideline (eTable 4 in the Supplement).^25^

### Data Sources

Data for this study were obtained from 8 health care databases housed at ICES.^26^ The following data sets were linked using unique encoded identifiers and analyzed at ICES: the Canadian Institute for Health Information Discharge Abstract Database, the ICES-derived Physician Database, the National Ambulatory Care Reporting System Database, the Ontario Drug Benefit Database, the Ontario Health Insurance Plan Database, the Ontario Laboratories Information System Database, the Ontario Mental Health Reporting System Database, and the Registered Persons Database. Data on hospital admissions and diagnoses were coded by trained personnel using the *International Statistical Classification of Diseases and Related Health Problems, Tenth Revision* system; personnel only consider physician-recorded diagnoses in a patient’s medical record when assigning codes and do not review or interpret symptoms or test results. These databases have been used before to study prescription drug safety.^27,28,29,30,31^ Except for prescriber data (7.0% missing, defined as a separate category) and neighborhood income quintile (0.4% missing, recorded as the middle quintile), the databases were complete for all variables in this study. The only reason for loss to follow-up was emigration from the province, which is less than 0.5% per year on average.^32^ The codes used to ascertain comorbidities and outcomes are detailed in eTable 5 in the Supplement.

### Study Cohort

We assembled a cohort of adults 66 years and older with an eGFR of less than 30 mL/min per 1.73 m^2^ (excluding patients receiving dialysis and kidney transplant recipients) who received a new prescription for a single oral fluoroquinolone (ciprofloxacin, levofloxacin, or norfloxacin) dispensed from an outpatient pharmacy between January 1, 2008, and March 17, 2020. The dispense date served as the date of cohort entry (ie, the index date). The age threshold of 66 years and older was used to ensure all patients had at least 1 year of prescription drug coverage before the index date. We restricted the cohort to patients with at least 1 serum creatinine measurement in the period from 1 year to 7 days before the index date (we excluded those with measurements within the 7-day period before the index date because these creatinine values may be elevated owing to acute infection).

To ensure patients were new fluoroquinolone users, we excluded those with evidence of fluoroquinolone use in the 180-day period before the index date. Ontario health care databases do not contain in-hospital treatment information, which may introduce an immeasurable time bias in pharmacoepidemiologic studies.^33^ To ensure study antibiotic treatments were initiated in an outpatient setting and to reduce the immeasurable time bias,^33^ we excluded patients discharged from the hospital or emergency department within 2 days before the index date (in Ontario, patients who start a fluoroquinolone prescription during a hospital admission would have their outpatient prescription dispensed on the same day or the day after hospital discharge). To ensure generalizability to usual prescribing, we excluded patients prescribed nonstandard doses (ie, ciprofloxacin, <500 mg/d or >1000 mg/d; levofloxacin, <250 mg/d or >750 mg/d; and norfloxacin, <400 mg/d or >800 mg/d). We also excluded patients prescribed a topical fluoroquinolone. Each patient could only enter the cohort once. This study had 80% power to detect a relative risk increase of at least a 58% for the primary outcome (corresponding to a risk ratio [RR] of 1.58; 2-sided α = .05; an incidence of 1% of the primary outcome in patients initiating treatment with a lower-dose fluoroquinolone) based on a feasibility analysis results.

### Baseline Characteristics

We determined the baseline eGFR using the most recent outpatient serum creatinine measurement recorded in the period from 1 year to 7 days before the index date (serum creatinine level was assessed using a method calibrated to isotope dilution mass spectroscopy).^34^ We calculated the eGFR using the Chronic Kidney Disease Epidemiology equation without applying the inflation factor for Black race^34^; Ontarians with African ancestry represented less than 5% of the population in 2016.^35^ Justification for this equation is provided in eTable 6 in the Supplement.^34^ In Ontario, most older adults have at least 1 outpatient serum creatinine level measured in routine care each year. We have shown that single creatinine values measured in the outpatient setting provide accurate staging of CKD.^36^ We assessed comorbidities in the 5-year period before cohort entry and health care use in the 1-year period before cohort entry. We used a 120-day look-back period to ascertain use of other prescription drugs because the Ontario Drug Benefit program allows a maximum prescription duration of 100 days.

### Exposed and Comparator Groups

The exposure in this study was a new prescription for a single higher-dose oral fluoroquinolone, defined as ciprofloxacin, 501 to 1000 mg/d; levofloxacin, 501 to 750 mg/d; or norfloxacin, 401 to 800 mg/d. The comparator group consisted of patients with a new prescription for a single lower-dose oral fluoroquinolone, defined as ciprofloxacin, 500 mg/d; levofloxacin, 250 to 500 mg/d; or norfloxacin, 400 mg/d. The dose thresholds were chosen based on current prescribing guidelines for patients with advanced CKD (eTable 1 in the Supplement) and the distribution of doses for these medications in our region (eTable 7 in the Supplement). There were too few patients with ofloxacin prescriptions in our region to be included in the study.

### Outcomes

We prespecified all primary and secondary outcomes. We used a 14-day time frame for all outcomes because fluoroquinolone-associated adverse events usually occur within days of treatment initiation (eTable 3 in the Supplement).^4^ Whenever possible, we used algorithms with a high positive predictive value to identify primary and secondary outcomes. Information on diagnostic codes and their validation and interpretation are provided in eTable 8 in the Supplement.

We defined the primary outcome as a composite of a hospital visit (ie, an emergency department visit or a hospital admission) with nervous system and/or psychiatric disorders, hypoglycemia, or collagen-associated events within 14 days of starting a new fluoroquinolone prescription. We chose to combine these events in a composite outcome to increase statistical power. Fluoroquinolones have been linked to these events in the general population and in patients with CKD (eTable 3 in the Supplement).^4,5,6,7,8,9,11,12,13^ We defined nervous system and/or psychiatric disorders as a diagnosis of altered mental status (ie, delirium, disorientation, transient alteration of awareness, agitation and nervousness, somnolence, and/or dizziness and giddiness) or peripheral neuropathy. We defined a collagen-associated event as a diagnosis of Achilles tendon rupture or abdominal aortic aneurysm rupture. Fluoroquinolone can damage connective tissue and collagen throughout the body and cause these events.^37,38^ Retinal detachment was not included in the primary outcome because an FDA update in May 2017 stated available data did not support a causal association between fluoroquinolones and retinal detachment (we examined this event as a secondary outcome).^19^ The secondary outcomes included components of the primary composite outcome examined separately, all-cause hospitalization, all-cause mortality, sudden cardiac death, a hospital visit with sepsis, and a hospital visit with retinal detachment or other tendinopathy.

### Statistical Analysis

Analyses were performed from January 1 to April 30, 2021, using SAS, version 9.4 (SAS Institute Inc). We used inverse probability of treatment weighting on the propensity score to balance the exposure and comparator groups on 147 baseline health indicators.^39,40,41^ We estimated the propensity score using multivariable logistic regression with 121 covariates chosen a priori (defined in eTable 9 in the Supplement) because they are known confounders or risk factors for adverse drug events.^41,42,43^ We weighted patients in the comparator group using average treatment effect for the treated weights defined as the propensity score divided by 1 minus the propensity score, with patients in the exposed group receiving weights of 1.^39,40,41^ This method produces a weighted pseudosample of patients in the comparator group with a similar distribution of measured covariates as the exposed group.^39,40^ We compared between-group differences on baseline characteristics using standardized differences in both the unweighted and weighted samples (differences >10% were considered meaningful).^44^ We obtained weighted RRs and 95% CIs using modified Poisson regression^45^ and weighted risk differences and 95% CIs using a binomial regression model with an identity link function.

To comply with ICES privacy regulations to minimize the risk of identification, specific values of cells with 5 or fewer patients were suppressed (reported as <6). We interpreted 2-tailed *P* < .05 as statistically significant.

### Additional Analysis

We conducted the following 4 post hoc sensitivity analyses to assess the main results: (1) a survival analysis (with 14-day follow-up censoring on death) that met the proportional hazards assumption (nonsignificant high dose × follow-up time interaction term; *P* = .92); (2) an E-value analysis to assess the extent of unmeasured confounding that would be required to negate the observed results^46^; (3) an analysis using a negative control outcome,^47^ defined as a hospital admission with heart failure; and (4) an analysis using a fine stratification weighting method to balance comparison groups on baseline health indicators. In contrast to the method of inverse probability of treatment weighting, the fine stratification weighting technique is less sensitive to the influence of extreme weights.^48^

## Results

### Patients

The flow diagram for the cohort build is presented in eFigure 1 in the Supplement. We studied 11 917 older adults with an eGFR of less than 30 mL/min per 1.73 m^2^ (median age, 83 years [IQR, 77-89 years]; 7438 women [62.4%] and 4479 men [37.6%] as self-reported in the Registered Persons Database; median eGFR, 25 [IQR, 21-28] mL/min/1.73 m^2^;926 [7.7%] had an eGFR of <15 mL/min per 1.73 m^2^) who were newly dispensed a fluoroquinolone at an outpatient pharmacy. The outpatient serum creatinine level to estimate baseline eGFR was measured a median of 54 (IQR, 22-115) days before cohort entry. Of 11 917 patients, 7614 (63.9%) were dispensed ciprofloxacin, 2492 (20.9%) were dispensed levofloxacin, and 1811 (15.2%) were dispensed norfloxacin. The median prescribed dose was 500 (range, 500-1000) mg/d for ciprofloxacin, 500 (range, 250-750) mg/d for levofloxacin, and 800 (range, 400-800) mg/d for norfloxacin (eTable 7 in the Supplement). Fluoroquinolone prescriptions were written by 5838 unique physicians and dispensed by 3582 unique pharmacies.

Fluoroquinolones were prescribed at higher than recommended doses in 5482 of 11 917 patients (46.0%); 6435 patients (54.0%) received a lower-dose fluoroquinolone. The median doses and days of continuous dispensing for each antibiotic in those prescribed higher vs lower doses is shown in eTable 10 in the Supplement. Characteristics of patients who started at higher vs lower doses are shown in Table 1 (the full set of 147 characteristics is shown in eTable 11 in the Supplement). Before any weighting, almost all indications for fluoroquinolone treatment were balanced between the 2 groups (high vs low dose). The proportion of patients with a recorded diagnosis of urinary tract infection (998 of 5482 [18.2%] vs 1266 of 6435 [19.7%]), prosthetic joint infection (766 of 5482 [14.0%] vs 854 of 6435 [13.3%]), and other bacterial infections (2117 of 5482 [38.6%] vs 2618 of 6435 [40.7%]) (Table 1 and eTable 11 in the Supplement) was similar between the high- and low-dose groups. We observed modest differences in the proportion of patients with a recorded diagnosis of community-acquired pneumonia (472 of 5482 [8.6%] vs 758 of 6435 [11.8%]) (Table 1 and eTable 11 in the Supplement). After weighting, the 2 groups were balanced on all 147 variables, including the prescriber type, recorded indication for fluoroquinolone use (ie, urinary tract infection, community-acquired pneumonia, prosthetic joint infection, and other bacterial infections), comorbidities, baseline eGFR, and concurrent medications (eTable 11 in the Supplement).

**Table 1.** Baseline Characteristics of Older Adults With Advanced Chronic Kidney Disease Newly Prescribed a Fluoroquinolone in Ontario, Canada, 2008 to 2020^a^

Characteristic	Unweighted data (n = 11 917)			Weighted data (n = 10 998)^b^		
	Fluoroquinolone dose		Standardized difference, %^c^	Fluoroquinolone dose^c^		Standardized difference, %^c^
	Higher (n = 5482)	Lower (n = 6435)		Higher (n = 5482)	Lower (n = 5516)	
Age, mean (SD), y	82 (8)	83 (8)	23	82 (8)	82 (8)	0
Sex						
Women	3287 (60.0)	4151 (64.5)	9	3287 (60.0)	3296 (59.7)	0
Men	2220 (40.2)	2284 (35.5)	9	2220 (40.2)	2219 (40.2)	0
Residence						
Urban	4846 (88.4)	5724 (89.0)	2	4846 (88.4)	4866 (88.2)	1
Rural	636 (11.6)	711 (11.0)	2	636 (11.6)	650 (11.8)	1
Long-term care	697 (12.7)	1450 (22.5)	26	697 (12.7)	686 (12.4)	1
Income quintile^d^						
First (lowest)	1244 (22.7)	1563 (24.3)	4	1244 (22.7)	1265 (22.9)	0
Second	1224 (22.3)	1434 (22.3)	0	1224 (22.3)	1225 (22.2)	0
Third (middle)	1147 (20.9)	1274 (19.8)	3	1147 (20.9)	1164 (21.1)	0
Fourth	1001 (18.3)	1138 (17.7)	2	1001 (18.3)	994 (18.0)	1
Fifth (highest)	868 (15.7)	1026 (15.9)	0	868 (15.7)	869 (15.7)	0
eGFR, mean (SD), mL/min per 1.73 m^2^^e^	23.9 (5.0)	23.3 (5.2)	12	23.9 (5.0)	23.9 (4.6)	0
Fluoroquinolone prescriber						
General clinician	4234 (77.2)	5120 (79.6)	6	4234 (77.2)	4241 (76.9)	1
Nephrologist	73 (1.3)	293 (4.5)	20	73 (1.3)	73 (1.3)	0
Internist	42 (0.8)	94 (1.5)	7	42 (0.8)	44 (0.8)	0
Urologist	402 (7.3)	177 (2.8)	21	402 (7.3)	427 (7.7)	2
Other	343 (6.3)	263 (4.1)	10	343 (6.3)	346 (6.3)	0
Missing	388 (7.1)	488 (7.6)	2	388 (7.1)	385 (7.0)	4
Comorbidities^f^						
Bipolar disorder	136 (2.5)	158 (2.5)	0	136 (2.5)	128 (2.3)	1
Coronary artery disease	2264 (41.3)	2725 (42.3)	2	2264 (41.3)	2306 (41.8)	1
Dementia	1344 (24.5)	2158 (33.5)	20	1344 (24.5)	1350 (24.5)	0
Hypertension	5036 (91.9)	5944 (92.4)	2	5036 (91.9)	5096 (92.4)	2
Depression	532 (9.7)	679 (10.5)	3	532 (9.7)	544 (9.9)	1
Diabetes	2107 (38.4)	2353 (36.6)	4	2107 (38.4)	2157 (39.1)	1
Urinary tract infection	998 (18.2)	1266 (19.7)	4	998 (18.2)	1030 (18.7)	1
Prosthetic joint infection	766 (14.0)	854 (13.3)	2	766 (14.0)	764 (13.9)	0
Community acquired pneumonia	472 (8.6)	758 (11.8)	11	472 (8.6)	470 (8.5)	0
Other bacterial infections	2117 (38.6)	2618 (40.7)	4	2117 (38.6)	2145 (38.9)	1
Charlson Comorbidity Index, mean (SD)^g^	3.2 (1.9)	3.4 (1.9)	6	3.2 (1.9)	3.2 (1.8)	1
Tests^h^						
Chest radiography	195 (3.5)	452 (7.0)	15	195 (3.5)	318 (5.8)	10
Urine culture	981 (17.9)	1081 (16.8)	3	981 (17.9)	936 (17.0)	2
Medication use^i^						
Statins	3266 (59.6)	3646 (56.7)	6	3266 (59.6)	3330 (60.4)	2
Opioids	1186 (21.6)	1453 (22.6)	2	1186 (21.6)	1214 (22.0)	1
Benzodiazepines	953 (17.4)	1125 (17.5)	0	953 (17.4)	950 (17.2)	1

Abbreviation: eGFR, estimated glomerular filtration rate.

^a^ Unless specified otherwise, baseline characteristics were assessed on the date the patient filled the fluoroquinolone prescription (ie, the cohort entry date), and data are expressed as No. (%) of patients. Percentages have been rounded and may not total 100.

^b^ Weighted using inverse probability of treatment weighting based on propensity scores.

^c^ Indicates the difference between the groups divided by the pooled SD; a value greater than 10% is interpreted as a meaningful difference.^44^

^d^ Categorized into fifths of mean neighborhood income on the cohort entry date; missing data on this variable (0.4%) were recorded as the middle quintile.

^e^ Indicates the most recent eGFR measurement in the 365-day period before the cohort entry date (including the cohort entry date), calculated using the Chronic Kidney Disease Epidemiology equation without applying the inflation factor for Black race.^34^

^f^ Baseline comorbidities were assessed in the 5-year period before the cohort entry date.

^g^ Presence of kidney disease is a variable in the Charlson Comorbidity Index, which automatically results in all individuals receiving a minimum score of 2.

^h^ Administered in the 7 days before the cohort entry date.

^i^ Examined in the 120-day period before the cohort entry date (the Ontario Drug Benefit program dispenses a maximum 100-day supply).

### Study Outcomes

After weighting, the primary composite outcome (a hospital visit with nervous system and/or psychiatric disorders, hypoglycemia, or a collagen-associated event) occurred in 68 of 5482 patients (1.2%) treated with a higher-dose fluoroquinolone and in 47 of 5516 patients (0.9%) treated with a lower-dose fluoroquinolone (weighted RR, 1.45 [95% CI, 1.01-2.08]; weighted risk difference, 0.39% [95% CI, 0.01%-0.76%]; number needed to harm, 256 [95% CI, 132-10 000]). In patients who experienced the outcome, the median time from starting a fluoroquinolone prescription to the event was 6 (IQR, 4-9) days in those prescribed higher doses and 7 (IQR, 4-9) days in those prescribed lower doses.

When the components of the primary composite outcome were examined separately, a higher vs lower fluoroquinolone dose was associated with a significantly higher 14-day risk of a hospital visit with altered mental status (RR, 1.77 [95% CI, 1.18-2.65]) but not hypoglycemia (RR, 0.97 [95% CI, 0.45-2.10]). The number of collagen-associated events was too low to report this outcome separately, and no cases of peripheral neuropathy were observed within 14 days of starting a new fluoroquinolone prescription (Table 2). A higher vs lower fluoroquinolone dose was not associated with any of the other secondary outcomes (ie, a hospital visit with sepsis or retinal detachment, all-cause hospitalization, sudden cardiac death, or all-cause mortality [the number of tendinopathy events was too low to report this outcome]) (Table 2).

**Table 2.** Risk of Primary and Secondary Outcomes in Older Adults With Advanced Chronic Kidney Disease Within 14 Days of Starting a New Prescription for a Higher- vs Lower-Dose Fluoroquinolone

Outcome	Analysis group, No. (%)				RD (95% CI), %	NNH (95% CI)	RR (95% CI)
	Unweighted by fluoroquinolone dose		Weighted by fluoroquinolone dose^a^				
	Higher (n = 5482)	Lower (n = 6435)	Higher (n = 5482)	Lower (n = 5516)			
Primary^b^	68 (1.2)	67 (1.0)	68 (1.2)	47 (0.9)	0.39 (0.01 to 0.76)	256 (132 to 10 000)	1.45 (1.01 to 2.08)
Secondary^c^							
Hospital visit with altered mental status	57 (1.0)	50 (0.8)	57 (1.0)	32 (0.6)	0.45 (0.13 to 0.78)	222 (128 to 769)	1.77 (1.18 to 2.65)
Hospital visit with hypoglycemia	13 (0.2)	17 (0.3)	13 (0.2)	14 (0.3)	−0.01 (−0.19 to 0.18)	NA	0.97 (0.45 to 2.10)
Hospital visit with sepsis	19 (0.3)	28 (0.4)	19 (0.3)	23 (0.4)	−0.08 (−0.34 to 0.19)	NA	0.82 (0.42 to 1.61)
Hospital visit with retinal detachment	15 (0.3)	12 (0.2)	15 (0.3)	8 (0.1)	0.12 (−0.05 to 0.29)	NA	1.78 (0.77 to 4.09)
All-cause hospitalization	336 (6.1)	405 (6.3)	336 (6.1)	317 (5.7)	0.39 (−0.52 to 1.30)	NA	1.07 (0.92 to 1.24)
All-cause mortality	88 (1.6)	166 (2.6)	88 (1.6)	86 (1.5)	0.04 (−0.39 to 0.47)	NA	1.02 (0.78 to 1.34)
Sudden cardiac death	26 (0.5)	48 (0.7)	26 (0.5)	27 (0.5)	−0.01 (−0.25 to 0.23)	NA	0.97 (0.59 to 1.61)

Abbreviations: NA, not applicable; NNH, number needed to harm; RD, risk difference; RR, risk ratio.

^a^ Inverse probability of treatment weighting on the propensity score was used to balance comparison groups on indicators of baseline health.^39,40,41^

^b^ Hospital visit with nervous system and/or psychiatric disorders, hypoglycemia, or a collagen-associated event.

^c^ The numbers of collagen-associated events and other tendinopathy events were too low to present these outcomes separately. No cases of peripheral neuropathy were observed within 14 days of starting a new fluoroquinolone prescription.

### Additional Analysis

The results of the primary analysis were supported by the 4 post hoc sensitivity analyses. First, results of the survival analysis were consistent when the data were analyzed using a Cox proportional hazards regression (eTable 12 in the Supplement). Second, the e-values for the RR were 2.26 and lower confidence bound for the primary outcome was 1.11 (eFigure 2 in the Supplement). Third, the risk of heart failure, a negative control outcome, was not significant (eTable 13 in the Supplement). Last, the results were consistent when fine stratification weights were used to balance comparison groups on baseline health indicators (eTable 14 in the Supplement).

## Discussion

In this population-based cohort study of 11 917 adults 66 years and older with advanced CKD, 46.0% were prescribed a fluoroquinolone at a higher than recommended dose in routine care. A higher vs lower fluoroquinolone dose was associated with a slightly higher 14-day risk of a hospital visit with nervous system and/or psychiatric disorders, hypoglycemia, or a collagen-associated event. Altered mental status was the most common adverse event. The mechanism behind fluoroquinolone-associated nervous system and/or psychiatric disorders is still not fully understood, but it has been hypothesized that fluoroquinolones may act as *N*-methyl-d-aspartate (NMDA) receptor agonists and partly as γ-aminobutyric acid A receptor antagonists.^49,50^ The absolute chance of experiencing a serious adverse event was low; for every 256 patients with advanced CKD prescribed a higher- vs lower-dose fluoroquinolone, approximately 1 was hospitalized with a serious adverse event. The results were consistent in several sensitivity analyses.

We conducted this study to address an evidence gap in safe fluoroquinolone prescribing in patients with advanced CKD. Our findings support current prescribing guidelines for patients with advanced CKD (eTable 1 in the Supplement). Our findings demonstrate that fluoroquinolones are prescribed at higher doses to nearly one-half the patients in this group. These results were consistent with 30 case reports of patients with CKD who developed toxic effects,^23^ often after initiation of a higher-dose fluoroquinolone prescription (eTable 3 in the Supplement). This finding also confirms results of a population-based retrospective time series analysis conducted in Ontario from 2003 to 2010^22^ that showed 54% of older adults with an eGFR of less than 30 mL/min/1.73 m^2^ were prescribed a higher ciprofloxacin dose. Our findings highlight that fluoroquinolones are being prescribed at higher than recommended doses, putting patients at higher risk of toxic effects.

### Strengths and Limitations

Our study has several strengths. To our knowledge, this is the first population-based study designed to assess the risk of adverse events associated with starting a higher- vs lower-dose fluoroquinolone prescription in patients with advanced CKD not receiving dialysis. The study was conducted in the setting of usual clinical care. It included a representative sample of older adults with advanced CKD in Ontario, Canada, where all residents 66 years and older have universal prescription drug coverage. We were able to produce comparison groups that were balanced on 147 baseline characteristics after using inverse probability of treatment weighting. We conducted several sensitivity analyses, and all results supported the main findings.

This study also has some limitations. First, although we used a robust statistical technique to control for confounding by indication and the results were consistent in sensitivity analyses, residual confounding cannot be ruled out. The e-values for the lower confidence bound for the primary outcome were small, which implies that little unmeasured confounding would be needed to reduce the observed association or its 95% CI to the null. Second, despite the use of highly accurate information on fluoroquinolone dispensing, administrative data cannot provide information on the proportion of patients who took their pills as prescribed. Third, we studied patients 66 years and older with advanced CKD, so our findings may not apply to younger patients or those receiving dialysis. Fourth, we examined renally excreted fluoroquinolones as a class, and there were too few prescriptions to perform independent analyses by fluoroquinolone type. Fifth, fluoroquinolone dosing can vary with the type of infection (eTable 1B in the Supplement); however, we could not perform subgroup analysis by infection type owing to small sample sizes. Sixth, misclassification of some study outcomes is expected for codes with low sensitivity, which would also underestimate the number of events (eTable 8 in the Supplement); however, differential misclassification between exposure groups is unlikely. Seventh, the small number of collagen-associated events and other tendinopathies precluded further analysis of these outcomes. Eighth, 926 of 11 917 patients (7.7%) had an eGFR of less than 15 mL/min/1.73 m^2^, and this small sample precluded any meaningful comparisons between CKD stages. Ninth, much of the data regarding adverse effects are derived from passive reporting systems and small observational studies, which are prone to confounding. Therefore, we chose to include adverse events reported in population-based drug safety studies and meta-analyses of randomized clinical trials and observational studies that resulted in FDA safety warnings. Although we believe that this algorithm for identifying fluoroquinolone-related adverse events is highly specific and robust, we may have missed some outcomes reported in case reports or pharmacovigilance reporting systems and some adverse events that did not result in safety warnings. Last, the magnitude of the risk and fragility of the statistical significance of many of the results may compromise the clinical relevance of our findings. Our study had 80% power to detect a relative risk increase of at least 58% for the primary outcome based on a feasibility analysis results. Although our study requires replication before definitive conclusions can be reached, the magnitude of the risk will likely be small. We did not consider fluoroquinolone effectiveness in this study, and in some cases, physicians may decide to prescribe a higher-dose fluoroquinolone because the potential benefits outweigh the risk.

## Conclusions

The findings of this cohort study suggest that fluoroquinolones should be prescribed cautiously and at lower doses among older adults with advanced CKD. All patients should be advised to watch for signs of altered mental status with fluoroquinolone use.

## Notes

Supplement. **eTable 1.** Recommended Dose of Oral Ciprofloxacin, Levofloxacin, and Norfloxacin Based on a Patient’s Kidney Function: Guidelines From UpToDate and the Product Monograph **eTable 2.** Literature Search **eTable 3.** Summary of Studies of Fluoroquinolone-Associated Adverse Events in Patients With Chronic Kidney Disease **eTable 4.** Checklist of Recommendations for Reporting of Observational Studies Using the Reporting of Studies Conducted Using Observational Routinely Collected Health Data (RECORD) Guidelines **eTable 5.** Coding Definitions for Demographic and Comorbid Conditions **eTable 6.** Justification for Using the CKD-EPI Equation to Estimate Patients’ Glomerular Filtration Rate in This Study **eTable 7.** Median Dose of Fluoroquinolone Dispensed to Adults Aged 66 and Older With an Estimated Glomerular Filtration Rate <30 mL/min/1.73 m^2^ in Ontario, Canada (2008 to 2020) **eTable 8.** Operating Characteristics of Hospital Diagnosis Codes Used to Define the Primary and Secondary Outcomes **eTable 9.** Variables Included in the Propensity Score Model **eTable 10.** Dose and Duration of Continuous Fluoroquinolone Dispensing in Older Adults With Advanced Chronic Kidney Disease Newly Prescribed a Fluoroquinolone in Ontario, Canada (2008-2020) **eTable 11.** Baseline Characteristics of Older Adults With Advanced Chronic Kidney Disease Newly Prescribed a Fluoroquinolone in Ontario, Canada (2008-2020) **eTable 12.** Post Hoc Survival Analysis in Older Adults With Advanced Chronic Kidney Disease Within 14 Days of Starting a New Prescription for a Higher- Vs Lower-Dose Fluoroquinolone: Risk of a Hospital Visit With Nervous System and/or Psychiatric Disorders, Hypoglycemia, or a Collagen-Associated Event **eTable 13.** Risk of Heart Failure in Older Adults With Advanced Chronic Kidney Disease Within 14 Days of Starting a New Prescription for a Higher- vs Lower-Dose Fluoroquinolone **eTable 14.** Risk of a Hospital Visit With Nervous System and/or Psychiatric Disorders, Hypoglycemia, or a Collagen-Associated Event in Older Adults With Advanced Chronic Kidney Disease Within 14 Days of Starting a New Prescription for a Higher- vs Lower-Dose Fluoroquinolone Using Fine Stratification Weighting **eFigure 1.** Flow Diagram of Cohort Build **eFigure 2.** e-Value Analysis to Assess the Extent of Unmeasured Confounding That Would Be Required to Negate the Observed Results
eReferences
