# Supplementary material for: Association of Higher-Dose Fluoroquinolone Therapy With Serious Adverse Events in Older Adults With Advanced Chronic Kidney Disease
Source: JAMA Netw Open. 2022 Aug 2;5(8):e2224892. doi: 10.1001/jamanetworkopen.2022.24892 (PMC9346548; doi:10.1001/jamanetworkopen.2022.24892)
Supplement: Supplement. — eTable 1. Recommended Dose of Oral Ciprofloxacin, Levofloxacin, and Norfloxacin Based on a Patient’s Kidney Function: Guidelines From UpToDate and the Product Monograph eTable 2. Literature Search eTable 3. Summary of Studies of Fluoroquinolone-Associated Adverse Events in Patients With Chronic Kidney Disease eTable 4. Checklist of Recommendations for Reporting of Observational Studies Using the Reporting of Studies Conducted Using Observational Routinely Collected Health Data (RECORD) Guidelines eTable 5. Coding Definitions for Demographic and Comorbid Conditions eTable 6. Justification for Using the CKD-EPI Equation to Estimate Patients’ Glomerular Filtration Rate in This Study eTable 7. Median Dose of Fluoroquinolone Dispensed to Adults Aged 66 and Older With an Estimated Glomerular Filtration Rate <30 mL/min/1.73 m2 in Ontario, Canada (2008 to 2020) eTable 8. Operating Characteristics of Hospital Diagnosis Codes Used to Define the Primary and Secondary Outcomes eTable 9. Variables Included in the Propensity Score Model eTable 10. Dose and Duration of Continuous Fluoroquinolone Dispensing in Older Adults With Advanced Chronic Kidney Disease Newly Prescribed a Fluoroquinolone in Ontario, Canada (2008-2020) eTable 11. Baseline Characteristics of Older Adults With Advanced Chronic Kidney Disease Newly Prescribed a Fluoroquinolone in Ontario, Canada (2008-2020) eTable 12. Post Hoc Survival Analysis in Older Adults With Advanced Chronic Kidney Disease Within 14 Days of Starting a New Prescription for a Higher- Vs Lower-Dose Fluoroquinolone: Risk of a Hospital Visit With Nervous System and/or Psychiatric Disorders, Hypoglycemia, or a Collagen-Associated Event eTable 13. Risk of Heart Failure in Older Adults With Advanced Chronic Kidney Disease Within 14 Days of Starting a New Prescription for a Higher- vs Lower-Dose Fluoroquinolone eTable 14. Risk of a Hospital Visit With Nervous System and/or Psychiatric Disorders, Hypoglycemia, or a Collagen-Associated Event in Older [file jamanetwopen-e2224892-s001.pdf]

Supplementary Online Content

Muanda FT, Sood MM, Weir MA, et al. Association of higher-dose fluoroquinolone therapy with serious adverse events in older adults with advanced chronic kidney disease. *JAMA Netw Open*. 2022;5(8):e2224892.
doi:10.1001/jamanetworkopen.2022.24892

eTable 1. Recommended Dose of Oral Ciprofloxacin, Levofloxacin, and Norfloxacin Based on a Patient's Kidney Function: Guidelines From UpToDate and the Product Monograph
eTable 2. Literature Search
eTable 3. Summary of Studies of Fluoroquinolone-Associated Adverse Events in Patients With Chronic Kidney Disease
eTable 4. Checklist of Recommendations for Reporting of Observational Studies Using the Reporting of Studies Conducted Using Observational Routinely Collected Health Data (RECORD) Guidelines
eTable 5. Coding Definitions for Demographic and Comorbid Conditions
eTable 6. Justification for Using the CKD-EPI Equation to Estimate Patients' Glomerular Filtration Rate in This Study
eTable 7. Median Dose of Fluoroquinolone Dispensed to Adults Aged 66 and Older With an Estimated Glomerular Filtration Rate <30 mL/min/1.73 m^2 in Ontario, Canada (2008 to 2020)
eTable 8. Operating Characteristics of Hospital Diagnosis Codes Used to Define the Primary and Secondary Outcomes
eTable 9. Variables Included in the Propensity Score Model
eTable 10. Dose and Duration of Continuous Fluoroquinolone Dispensing in Older Adults With Advanced Chronic Kidney Disease Newly Prescribed a Fluoroquinolone in Ontario, Canada (2008-2020)
eTable 11. Baseline Characteristics of Older Adults With Advanced Chronic Kidney Disease Newly Prescribed a Fluoroquinolone in Ontario, Canada (2008-2020)
eTable 12. Post Hoc Survival Analysis in Older Adults With Advanced Chronic Kidney Disease Within 14 Days of Starting a New Prescription for a Higher- Vs Lower-Dose Fluoroquinolone: Risk of a Hospital Visit With Nervous System and/or Psychiatric Disorders, Hypoglycemia, or a Collagen-Associated Event
eTable 13. Risk of Heart Failure in Older Adults With Advanced Chronic Kidney Disease Within 14 Days of Starting a New Prescription for a Higher- vs Lower-Dose Fluoroquinolone
eTable 14. Risk of a Hospital Visit With Nervous System and/or Psychiatric Disorders, Hypoglycemia, or a Collagen-Associated Event in Older Adults With

© 2022 Muanda FT et al. *JAMA Network Open*.

Advanced Chronic Kidney Disease Within 14 Days of Starting a New Prescription for a Higher- vs Lower-Dose Fluoroquinolone Using Fine Stratification Weighting
eFigure 1. Flow Diagram of Cohort Build
eFigure 2. e-Value Analysis to Assess the Extent of Unmeasured Confounding That Would Be Required to Negate the Observed Results
eReferences

This supplementary material has been provided by the authors to give readers additional information about their work.

© 2022 Muanda FT et al. *JAMA Network Open*.

eTable 1. Recommended Dose of Oral Ciprofloxacin, Levofloxacin, and Norfloxacin Based on a Patient's Kidney Function: Guidelines From UpToDate and the Product Monograph

eTable 1a. Recommended dose of oral ciprofloxacin

UpToDate Guidelines[1]	Product Monograph[2]
**Creatinine clearance >50 to <130 mL/min**	**Creatinine clearance >50 to <130 mL/min**
Oral Immediate Release: 500-750 mg every 12h<br>Oral Extended Release: 1g every 24h	NA
**Creatinine clearance 30 to 50 mL/min**	**Creatinine clearance 31 to 60 mL/min/1.73 m^2**
Oral Immediate Release: 250-500 mg every 12h<br>Oral Extended Release: 1g every 24h	Maximum daily oral dose: 1000 mg
**Creatinine clearance <30 mL/min**	**Creatinine clearance ≤30 mL/min/1.73 m^2**
Oral Dose: 500mg every 24hrs<br>Oral Extended Release: 500 mg every 24h	Maximum daily oral dose: 500 mg

Abbreviations: NA, not available.

© 2022 Muanda FT et al. *JAMA Network Open*.

eTable 1b. Recommended dose of oral levofloxacin

UpToDate Guidelines[3]	Product Monograph[4]
**Creatinine clearance ≥ 50 mL/min**	**Creatinine clearance 50 to 80 mL/min**
No dosage adjustment required.	No dosage adjustment required.
**Creatinine clearance 20 to <50 mL/min**	**Creatinine clearance 20 to 49 mL/min**
If recommended dose is 250 mg every 24h: no dosage adjustment is required.<br><br>If recommended dose is 500 mg every 24h: 500mg initial dose, then 250 mg every 24hrs.<br><br>If recommended dose is 750 mg every 24h: 750 mg every 48hr.	Initial dose: 500 mg, subsequent dose: 250 mg every 24h (acute sinusitis, acute bacterial exacerbation of chronic bronchitis, community-acquired pneumonia, uncomplicated skin and skin structure infections, chronic bacterial prostatitis).<br><br>Initial dose: 750 mg, subsequent dose 750mg every 48h (complicated skin and skin structure infections/nosocomial pneumonia/ community acquired pneumonia /acute bacterial exacerbation of chronic bronchitis/acute sinusitis/complicated urinary tract infection/acute pyelonephritis).
**Creatinine clearance <20mL/min**	**Creatinine clearance 10 to 19 mL/min**
If recommended dose is 250 mg every 24h: 250 mg every 48h (except for uncomplicated urinary tract infection, where no dosage adjustment necessary).<br><br>If recommended dose 500 mg every 24h: 500 mg initial dose, then 250mg every 48 h.<br><br>If recommended dose is 750 mg every 24h: 750 mg initial dose, then 500mg every 48h.	Initial dose: 500 mg, subsequent dose 250 mg every 48h (acute sinusitis, acute bacterial exacerbation of chronic bronchitis, community-acquired pneumonia, uncomplicated skin structure infections/nosocomial pneumonia, chronic bacterial prostatitis)<br>Initial dose: 250 mg, subsequent dose 250mg every 48h (complicated urinary tract infection/acute pyelonephritis).<br><br>Initial dose: 750 mg, subsequent dose 500 mg every 48h (complicated skin structure infections/nosocomial pneumonia /nosocomial pneumonia/ community acquired pneumonia,/acute bacterial exacerbation of chronic bronchitis/acute sinusitis/complicated urinary tract infection/acute pyelonephritis).

© 2022 Muanda FT et al. *JAMA Network Open*.

eTable 1c. Recommended dose of oral norfloxacin

UpToDate Guidelines[5]	Product Monograph[6]
**Creatinine clearance >30 mL/min/1.73m^2**	**Creatinine clearance >30 mL/min/1.73m^2**
No dose adjustment required: 400 mg twice daily	No dose adjustment required: 400 mg twice daily
**Creatinine clearance rate ≤30mL/min/1.73m^2**	**Glomerular filtration rate <30 mL/min/1.73 m^2**
Recommended dose: 400 mg every 24h	Recommended dose: 400 mg every 24h

© 2022 Muanda FT et al. *JAMA Network Open*.

eTable 2. Literature Search

eTable 2a. A literature search in Medline (1946 to May 11, 2021)

1	exp Ciprofloxacin/ad, ae, pk, po, tu, to [Administration & Dosage, Adverse Effects, Pharmacokinetics, Poisoning, Therapeutic Use, Toxicity]
2	ciprofloxacin*.ti,ab,kw.
3	ciprofloxacin*.tw. /freq=2
4	exp Norfloxacin/ad, ae, pk, tu, to [Administration & Dosage, Adverse Effects, Pharmacokinetics, Therapeutic Use, Toxicity]
5	norfloxacin*.ti,ab,kw.
6	norfloxacin*.tw. /freq=2
7	exp Levofloxacin/ad, ae, pk, tu, to [Administration & Dosage, Adverse Effects, Pharmacokinetics, Therapeutic Use, Toxicity]
8	levofloxacin*.ti,ab,kw.
9	levofloxacin*.tw. /freq=2
10	1 or 2 or 3 or 4 or 5 or 6 or 7 or 8 or 9
11	(((chronic$ or progressive or diabetic) adj (kidney or renal or nephro$ or glomerul$)) or dialy$ or h?emodia$).mp. or ckd.tw. or esrd.tw. or ((diabet$.mp. or Disease Progression/ or Recurrence/) and nephropath$.mp.) or ur?emi$.mp. or m?croalbuminuri$.mp. or albuminuri$.mp. or proteinuri$.mp. or nephrosclerosis.mp. or glomerulosclerosis.mp. or glomerular sclerosis.mp. or *Glomerular Filtration Rate/ or (secondary adj2 hyperparathyroidism).mp. or ((tubulointerstitial or interstitial or renal or kidney) adj fibrosis).tw. or hyperphosphat?emia.tw. or vascular calcification$.tw. or alport$.mp. or denys- drash.mp. or glomerulopathy.tw. or hypoalbumin?emi$.mp. or multicystic kidney$.mp. or polycystic kidney$.mp. or cystic kidney$.mp. or calciphylaxis.mp. or tenckhoff.tw. or ((kidney or renal) adj (disease$ or failur$ or function$ or insufficienc$ or disorder$ or dysfunction or replacement)).mp. or ((kidney or renal) and (ckf or crd or crf or eskd or eskf or esrf or hyperparathyroidism or end-stage or endstage or eGFR)).mp. or (((kidney or renal) adj transplant$) and (candidates or wait$ list$)).tw. or ((sclerosi$ or fibrosi$ or fibrotic).mp. and ((ureteral obstruction or nephritis or glomerulonephritis or nephrop$).mp. or (obstruct$ and (kidney$ or renal or nephropathy)).tw.))
12	exp Renal Insufficiency, Chronic/co, dt, pc, th [Complications, Drug Therapy, Prevention & Control, Therapy]
13	exp Renal Replacement Therapy/
14	11 or 12 or 13
15	10 and 14
16	exp "drug-related side effects and adverse reactions"/ or adverse.ti,ab,kf. or side effect?.ti,ab,kf. or adverse effects.fs. or exp drug overdose/ or overdos*.ti,ab,kf. or exp drug misuse/ or misus*.ti,ab,kf. or exp substance-related disorders/ or abus*.ti,ab,kf. or exp pregnancy/ or pregnan*.ti,ab,kf. or exp pregnancy complications/ or exp lactation/ or exp lactation disorders/ or exp breast feeding/ or (exp milk, human/ and exp secretion/) or exp fertility/ or exp infertility/ or exp reproduction/ or exp fetus/ or exp embryonic structures/ or terat*.ti,ab,kf. or drug efficacy.ti,ab,kf. or therapeutic efficacy.ti,ab,kf. or drug withdrawal.ti,ab,kf. or exp medication errors/ or exp death/ or death*.ti,ab,kf. or fatal*.ti,ab,kf. or exp drug interactions/ or exp carcinogens/ or carcinogen*.ti,ab,kf. or mutagen*.ti,ab,kf. or exp "off-label use"/ or exp occupational exposure/ or toxicity.fs. or toxic*.ti,ab,kf. or pharmacotox*.ti,ab,kf. or neurotox*.ti,ab,kf. or cardiotox*.ti,ab,kf. or nephrotox*.ti,ab,kf. or immunotox*.ti,ab,kf. or hepatotox*.ti,ab,kf. or cytotox*.ti,ab,kf. or immunocytotox*.ti,ab,kf. or intoxicat*.ti,ab,kf. or exp "congenital, hereditary, and neonatal diseases and abnormalities"/ or drug treatment failure.ti,ab,kf. or drug toxicity.ti,ab,kf. or exp case

© 2022 Muanda FT et al. *JAMA Network Open*.

	report/ or case report?.ti,ab,kf. or exp environmental exposure/ or treatment contraindication.ti,ab,kf. or exp contraindications, drug/ or exp "wounds and injuries"/ or suicid*.ti,ab,kf. or exp poisoning/ or poisoning.fs. or exp drug tolerance/ or exp treatment failure/ or exp drug resistance/ or exp substance-related disorders/
17	15 and 16
18	limit 17 to (english language and humans)

eTable 2b. Literature search in Embase (1947 to May 12, 2021)

1	exp ciprofloxacin/ae, ct, ad, cm, do, dt, to, pv, tm [Adverse Drug Reaction, Clinical Trial, Drug Administration, Drug Comparison, Drug Dose, Drug Therapy, Drug Toxicity, Special Situation for Pharmacovigilance, Unexpected Outcome of Drug Treatment]
2	ciprofloxacin*.ti,ab,kw.
3	ciprofloxacin*.tw. /freq=2
4	exp norfloxacin/ae, ct, ad, cm, do, dt, to, pv, tm [Adverse Drug Reaction, Clinical Trial, Drug Administration, Drug Comparison, Drug Dose, Drug Therapy, Drug Toxicity, Special Situation for Pharmacovigilance, Unexpected Outcome of Drug Treatment]
5	norfloxacin*.ti,ab,kw.
6	norfloxacin*.tw. /freq=2
7	exp levofloxacin/ae, ct, ad, cm, do, dt, to, pv, tm [Adverse Drug Reaction, Clinical Trial, Drug Administration, Drug Comparison, Drug Dose, Drug Therapy, Drug Toxicity, Special Situation for Pharmacovigilance, Unexpected Outcome of Drug Treatment]
8	levofloxacin*.ti,ab,kw.
9	levofloxacin*.tw. /freq=2
10	(((chronic$ or progressive or diabetic) adj (kidney or renal or nephro$ or glomerul$)) or dialy$ or h?emodia$).mp. or ckd.tw. or esrd.tw. or ((diabet$.mp. or Disease Progression/ or Recurrence/) and nephropath$.mp.) or ur?emi$.mp. or m?croalbuminuri$.mp. or albuminuri$.mp. or proteinuri$.mp. or nephrosclerosis.mp. or glomerulosclerosis.mp. or glomerular sclerosis.mp. or *Glomerular Filtration Rate/ or (secondary adj2 hyperparathyroidism).mp. or ((tubulointerstitial or interstitial or renal or kidney) adj fibrosis).tw. or hyperphosphat?emia.tw. or vascular calcification$.tw. or alport$.mp. or denys- drash.mp. or glomerulopathy.tw. or hypoalbumin?emi$.mp. or multicystic kidney$.mp. or polycystic kidney$.mp. or cystic kidney$.mp. or calciphylaxis.mp. or tenckhoff.tw. or ((kidney or renal) adj (disease$ or failur$ or function$ or insufficienc$ or disorder$ or dysfunction or replacement)).mp. or ((kidney or renal) and (ckf or crd or crf or eskd or eskf or esrf or hyperparathyroidism or end-stage or endstage or eGFR)).mp. or (((kidney or renal) adj transplant$) and (candidates or wait$ list$)).tw. or ((sclerosi$ or fibrosi$ or fibrotic).mp. and ((ureteral obstruction or nephritis or glomerulonephritis or nephrop$).mp. or (obstruct$ and (kidney$ or renal or nephropathy)).tw.))
11	exp kidney disease/co, dm, dt, si, th [Complication, Disease Management, Drug Therapy, Side Effect, Therapy]
12	exp renal replacement therapy/ae [Adverse Drug Reaction]
13	10 or 11 or 12
14	exp adverse drug reaction/ or adverse.ti,ab,kw. or side effect?.ti,ab,kw. or side effect.fs. or exp drug overdose/ or overdos*.ti,ab,kw. or exp drug misuse/ or misus*.ti,ab,kw. or exp drug abuse/ or exp substance abuse/ or abus*.ti,ab,kw. or exp pregnancy/ or pregnan*.ti,ab,kw. or exp pregnancy complications/ or exp lactation/ or exp breast feeding/ or (exp milk human/ and exp secretion/) or exp fertility/ or exp infertility/ or exp reproduction/ or exp fetus/ or exp embryo/ or terat*.ti,ab,kw. or exp drug efficacy/ or exp drug withdrawal/ or exp medication error/ or

© 2022 Muanda FT et al. *JAMA Network Open*.

	exp death/ or death*.ti,ab,kw. or fatal*.ti,ab,kw. or exp drug interaction/ or exp carcinogenicity/ or carcinogen*.ti,ab,kw. or mutagen*.ti,ab,kw. or exp 'off label drug use'/ or exp occupational exposure/ or exp toxicity/ or toxic*.ti,ab,kw. or pharmacotox*.ti,ab,kw. or neurotox*.ti,ab,kw. or cardiotox*.ti,ab,kw. or nephrotox*.ti,ab,kw. or immunotox*.ti,ab,kw. or hepatotox*.ti,ab,kw. or cytotox*.ti,ab,kw. or immunocytotox*.ti,ab,kw. or exp intoxication/ or exp congenital disorder/ or exp drug treatment failure/ or exp case report/ or case report?.ti,ab,kw. or exp environmental exposure/ or exp treatment contraindication/ or exp drug contraindication/ or exp injury/ or suicid*.ti,ab,kw. or exp poisoning/ or exp drug tolerance/ or exp treatment failure/ or exp drug resistance/ or exp substance-related disorders/ or drug resistance.fs.
15	1 or 2 or 3 or 4 or 5 or 6 or 7 or 8 or 9
16	13 and 14 and 15
17	limit 16 to (human and english language)

© 2022 Muanda FT et al. *JAMA Network Open*.

Table 3. Summary of Studies of Fluoroquinolone-Associated Adverse Events in Patients With Chronic Kidney Disease

eTable 3a. Case Reports

Author/year of publication	Age/sex	FQ administered	FQ dose (mg/day)	Degree of CKD, eGFR, or CrCl ml/min/1.73m^2	Type of toxicity	Days to toxicity onset[a]	Treatment	Naranjo score[b]
Abdalla 2013[7]	72/male	Ciprofloxacin	1000	on hemodialysis	choreoathetosis	3 days	D/C drug	5
Stroud 2020[8]	56/female	Ciprofloxacin	500	11	delirium and hypoglycemia	1 day	D/C drug and HD	3
Schwalm 2003[9]	73/male	Levofloxacin	250	on hemodialysis	acute hepatitis	21 days	D/C drug	7
Abo-salem 2011[10]	92/female	IV Levofloxacin	750	on hemodialysis	Tdp arrhythmia	4 hours	D/C drug	7
Kawtharani 2016[11]	70/male	Ciprofloxacin	1000	A case of MCD/ SCr =1.3-1.6	tendinopathy	4 days	not mentioned	6
Denysenko 2011[12]	60/female	IV Ciprofloxacin	400	on hemodialysis	neurotoxicity and catatonia	1 day	D/C drug	2
Martin 2020[13]	76/male	Ciprofloxacin	1500	Stage 2 CKD	acute renal failure	9 days	D/C drug and supportive care for AKI	6
Sedlacek 2006[14]	90/female	Ciprofloxacin	1500	Stage 3 CKD	acute renal failure secondary to ciprofloxacin-induced crystal nephropathy	8 days	D/C drug	7
Reece 1996[15]	41/male	Ciprofloxacin	1000	SCr =1.8	AIN	3 days	D/C drug, prednisolone administration	7
Striano 2007[16]	63/male	IV Ciprofloxacin	200	SCr =4	myoclonus	2 days	D/C drug and lorazepam	7
Matoi 2021[17]	73/male	IV Ciprofloxacin	400	on hemodialysis	fatal hypoglycemia	7 days	not mentioned	
Marti 1998[18]	55/male	Ciprofloxacin	500	22	tendinopathy	3 days	not mentioned	5

© 2022 Muanda FT et al. *JAMA Network Open*.

Author/year of publication	Age/sex	FQ administered	FQ dose (mg/day)	Degree of CKD, eGFR, or CrCl ml/min/1.73m^2	Type of toxicity	Days to toxicity onset[a]	Treatment	Naranjo score[b]
	58/female	Ciprofloxacin	500	40		5 days		5
	37/male	Ciprofloxacin	500	on hemodialysis		60 days		5
Takeda 2012[19]	74/male	Levofloxacin	400	on hemodialysis	tendinopathy	8 days	D/C drug	7
	62/male	Levofloxacin	500 mg initial, then 250mg every 48 hrs	on hemodialysis		2 days	D/C drug	7
	76/male	Levofloxacin	500 mg initial, then 250mg every 48 hrs	on hemodialysis		3 days	D/C drug	7
	66/male	Levofloxacin	500 mg initial, then 250mg every 48 hrs	on hemodialysis		1 day	D/C drug	7
Idrees 2019[20]	82/female	IV Levofloxacin	750	on hemodialysis	myoclonus	1 day	D/C drug and HD	4
Gkoufa 2020[21]	84/male	Ciprofloxacin	1000	37	Henoch-Schönlein purpura	2 days	D/C drug, Hydration and daily methylprednisolone	5
Korzets 2006[22]	68/male	Levofloxacin	250	30	rhabdomyolysis	10 days	D/C drug	7
Kato 2011[23]	63/female	Levofloxacin	500 mg every two days	8	tendinopathy	3 days	D/C drug	7
Nishikubo 2019[24]	68/male	Levofloxacin	500	SCr =2.5	neurotoxicity	11 days	D/C drug and HD	7
Patil 2020[25]	58/female	Levofloxacin	750	stage 5 CKD	hyperpigmented rash	2 days	not mentioned but medication had not been stopped	5
Majda 2020[26]	87/female	IV Levofloxacin	500	23	Hypoglycemia	3 days	glucose injection, medication had not been stopped	5
Kelesidis 2010[27]	65/female	Ciprofloxacin	500	Mentioned by the authors as a case of CKD	hypoglycemia	a few hours	dextrose and octreotide	5

© 2022 Muanda FT et al. *JAMA Network Open*.

Author/year of publication	Age/sex	FQ administered	FQ dose (mg/day)	Degree of CKD, eGFR, or CrCl ml/min/1.73m^2	Type of toxicity	Days to toxicity onset[a]	Treatment	Naranjo score[b]
Tsai 2014[28]	73/female	IV Levofloxacin	750	on hemodialysis	Tdp arrhythmia	same day of first dose adminis tration	cardioversion, replacing levofloxacin and HD	5
Kushner 2001[29]	75/female	Levofloxacin	500 mg on day 1 then 250	SCr =1.5	seizure	3 days	D/C drug and seizure management with magnesium and fosphenytoin	7
Parra-Riffo 2012[30]	72/male	Levofloxacin	250 mg every 48 hrs	on hemodialysis	hypoglycemia	during the 2nd week after first dose	glucose administration	7
Proietti 2011[31]	80/male	IV Levofloxacin	500	27	Tdp arrhythmia	2 days	management of arrhythmia and D/C drug	5

Abbreviations: CrCl, creatinine clearance; eGFR, estimated glomerular filtration rate; CKD, chronic kidney disease; FQ, fluoroquinolone, D/C discontinue; HD, hemodialysis; IV, intravenous; tdp, torsades de pointes; Minimal Change Disease, MCD; SCr, serum creatinine.

[a] The median [IQR] time to toxicity was 3 (2-8) days after a fluoroquinolone initiation in these case reports.

[b]Naranjo Adverse Drug Reaction Probability Interpretation: ≥ 9 = definite ADR, 5-8 = probable ADR, 1-4 = possible ADR, 0 = doubtful ADR.

© 2022 Muanda FT et al. *JAMA Network Open*.

eTable 3b: Retrospective cohort study

Author	Study description	Study procedure /exposure time	Results	Study limitation	Quality score[a]
Assimon 2021[32]	264,968 patients (Medicare beneficiaries) receiving in-center maintenance hemodialysis (mean age 61 years) newly prescribed a study antibiotic between 200-2016 in the United States	Respiratory fluoroquinolone (levofloxacin or moxifloxacin) vs amoxicillin-based (amoxicillin or amoxicillin with clavulanic acid) antibiotic treatment.<br>The primary outcome was sudden cardiac death within 5 days of outpatient initiation of a study antibiotic.<br>Fracture was considered as a negative control outcome	Respiratory fluoroquinolone vs amoxicillin-based antibiotic treatment was associated with a higher 5-day risk of sudden cardiac death (weighted HR, 1.95; 95% CI, 1.57-2.41)<br>Respiratory fluoroquinolone vs amoxicillin-based antibiotic treatment was not associated with the 5-day risk of fracture.	Residual confounding by indication	26

Abbreviations: HR, hazard ratio; CI, confidence interval.

[a] We evaluated the quality of the using the Modified Downs and Black checklist for the assessment of the methodological quality of this retrospective cohort study. We gave a score from 0 to 28, grouped into the following four quality levels: excellent (26 to 28), good (20-25), fair (15-19) and poor (14 or less).

© 2022 Muanda FT et al. *JAMA Network Open*.

eTable 4. Checklist of Recommendations for Reporting of Observational Studies Using the Reporting of Studies Conducted Using Observational Routinely Collected Health Data (RECORD) Guidelines

	Item No	Recommendation	Reported
**Title and abstract**	1	(a) Indicate the study's design with a commonly used term in the title or the abstract	Abstract
		(b) Provide in the abstract an informative and balanced summary of what was done and what was found	Abstract
**Introduction**			
Background/rationale	2	Explain the scientific background and rationale for the investigation being reported	Introduction
Objectives	3	State specific objectives, including any prespecified hypotheses	Introduction
**Methods**			
Study design	4	Present key elements of study design early in the paper	Methods - Study Design and Setting
Setting	5	Describe the setting, locations, and relevant dates, including periods of recruitment, exposure, follow-up, and data collection	Methods - Study Design and Setting; Method - Data Sources; Patient's selection and fluoroquinolone dosing
Participants	6	(a) Give the eligibility criteria, and the sources and methods of selection of participants. Describe methods of follow-up	Methods – patient selection and fluoroquinolone dosing; Supplemental eFigure 1
		(b) For matched studies, give matching criteria and number of exposed and unexposed	Statistical analysis; Results - Baseline Characteristics; Table 1; Supplemental eTable 11
Variables	7	Clearly define all outcomes, exposures, predictors, potential confounders, and	Methods - Data Sources; Methods - Outcomes.

© 2022 Muanda FT et al. *JAMA Network Open*.

	Item No	Recommendation	Reported
		effect modifiers. Give diagnostic criteria, if applicable	Supplemental eTable 5; Table 1
Data sources/measurement	8	For each variable of interest, give sources of data and details of methods of assessment (measurement). Describe comparability of assessment methods if there is more than one group	Methods - Data Sources. Supplemental eTable 5
Bias	9	Describe any efforts to address potential sources of bias	Methods – statistical analysis; additional analysis; Discussion
Study size	10	Explain how the study size was arrived at	Not applicable; use of existing health records
Quantitative variables	11	Explain how quantitative variables were handled in the analyses. If applicable, describe which groupings were chosen and why	Methods – Statistical Analysis and additional analysis
Statistical methods	12	(a) Describe all statistical methods, including those used to control for confounding	Methods – Statistical Analysis and additional analysis
		(b) Describe any methods used to examine subgroups and interactions	Methods – Statistical Analysis and additional analysis
		(c) Explain how missing data were addressed	Table 1; Supplemental eTable 11
		(d) If applicable, explain how loss to follow-up was addressed	Not applicable
		(e) Describe any sensitivity analyses	Methods – additional analyses;
**Results**			
Participants	13	(a) Report numbers of individuals at each stage of study--e.g. numbers potentially eligible, examined for eligibility, confirmed eligible, included in the study, completing follow-up, and analyzed	Results - Baseline Characteristics. Supplemental Figure 1; Table 1; Supplemental eTable 11

© 2022 Muanda FT et al. *JAMA Network Open*.

	Item No	Recommendation	Reported
		(b) Give reasons for non-participation at each stage	Supplemental eFigure 1
		(c) Consider use of a flow diagram	Supplemental eFigure 1
Descriptive data	14		
		(a) Give characteristics of study participants (e.g. demographic, clinical, social) and information on exposures and potential confounders	Results - Baseline Characteristics.<br>Table 1; Supplemental eTable 11
		(b) Indicate number of participants with missing data for each variable of interest	Methods - Data Sources; Table 1; Supplemental eTable 11
		(c) Summarize follow-up time (e.g. average and total amount)	Results - Primary Outcomes, Secondary Outcomes
Outcome data	15	Report numbers of outcome events or summary measures over time	Results - Primary Outcomes; Table 2;
Main results	16	(a) Give unadjusted estimates and, if applicable, confounder-adjusted estimates and their precision (e.g. 95% confidence interval). Make clear which confounders were adjusted for and why they were included	Results - Primary Outcomes, Secondary Outcomes.<br>Table 2
		(b) Report category boundaries when continuous variables were categorized	Not applicable
		(c) If relevant, consider translating estimates of relative risk into absolute risk for a meaningful time period	Table 2
Other analyses	17	Report other analyses done--e.g. analyses of subgroups and interactions, and sensitivity analyses	Results - Secondary Outcome.<br>Table 2 and Additional analyses, Supplemental eTable 12-14 and Supplemental eFigure 2

© 2022 Muanda FT et al. *JAMA Network Open*.

	Item No	Recommendation	Reported
**Discussion**			
Key results	18	Summarize key results with reference to study objectives	Discussion
Limitations	19	Discuss limitations of the study, taking into account sources of potential bias or imprecision. Discuss both direction and magnitude of any potential bias	Discussion
Interpretation	20	Give a cautious overall interpretation of results considering objectives, limitations, multiplicity of analyses, results from similar studies, and other relevant evidence	Discussion
Generalizability	21	Discuss the generalizability (external validity) of the study results	Discussion
**Other information**			
Funding	22	Give the source of funding and the role of the funders for the present study and, if applicable, for the original study on which the present article is based	Article Information

© 2022 Muanda FT et al. *JAMA Network Open*.

eTable 5. Coding Definitions for Demographic and Comorbid Conditions

Characteristic	Database	Codes
**Demographics**		
Age	RPDB	
Sex	RPDB	
Location of residence – Rural status	Statistics Canada	
Long-term care	ODB	LTC
Year of cohort entry	ODB	
Socioeconomic Status (Neighbourhood Income Quintile)	Statistics Canada	
LHIN[a]	RPDB	LHIN
Prescriber	ODB	
**Comorbidities (5 years prior the cohort entry date)**		
Acute kidney injury	CIHI-DAD	ICD-10: N17
Anxiety disorder and depression	CIHI-DAD	ICD-10: F063, F064, F204, F313, F314, F315, F32, F33, F341, F400, F401, F402, F408, F409, F410, F411, F412, F413, F418, F419, F420, F421, F422, F428, F429, F430, F431, F432
	OHIP	OHIP DX: 311
	OMHRS (DSM-IV)	29189, 29284, 29289, 29383, 29384, 29620, 29621, 29622, 29623, 29624, 29625, 29626, 29630, 29631, 29632, 29633, 29634, 29635, 29636, 30000, 30001, 30002, 30021, 30022, 30023, 30029, 30030, 30040, 30113
Anemia	CIHI-DAD	ICD10: D50 , D51 , D52 , D53 , D55 , D56 , D570 , D571, D58 , D59, D60 , D61 , D62 , D63, D64
	OHIP	OHIP dx: 280, 281, 282, 283, 284, 285
Bipolar disorder	CIHI-DAD	ICD-10: F300, F301, F302, F308, F309, F310, F311, F312, F313, F314, F315, F316, F317, F318, F319
	OHIP	OHIP DX: 296<br>OHIP FEE: Q020
	OMHRS (DSM-IV)	29600, 29601, 29602, 29603, 29604, 29605, 29606, 29640, 29641, 29642, 29643, 29644, 29645, 29646, 29650, 29651, 29652, 29653, 29654, 29655, 29656, 29660, 29661, 29662, 29663, 29664, 29665, 29666, 29670, 29680, 29689
Myocardial infarction	CIHI-DAD	ICD10: I21, I22
Atrial fibrillation/flutter	CIHI-DAD	ICD10: I48
Cancer	CIHI-DAD	ICD-10: 80003, 80006, 80013, 80023, 80033, 80043, 80102, 80103, 80106, 80113, 80123, 802, 803, 80413, 80423, 80433, 80443, 80453, 80502, 80503, 80513, 80523, 807, 808, 80903, 80913, 80923, 80933, 80943, 80953, 81103, 81202, 81203, 81213, 81223, 81233, 81243, 81303, 81402, 81403, 81406, 81413, 81423, 81433, 81443, 81453, 81473, 81503, 81513, 81523, 81533, 81543, 81553, 81603, 81613, 81623, 81703,

© 2022 Muanda FT et al. *JAMA Network Open*.

Characteristic	Database	Codes
		81713, 81803, 81903, 82003, 82013, 82102, 82103, 82113, 82203, 82213, 823, 82403, 82413, 82433, 82443, 82453, 82463, 82473, 82503, 82513, 82603, 82612, 82613, 82623, 82632, 82633, 82703, 82803, 82813, 82903, 83003, 83103, 83123, 83143, 83153, 83203, 83223, 83233, 83303, 83313, 83323, 83403, 83503, 83703, 83803, 83813, 83903, 84003, 84013, 84103, 84203, 84303, 84403, 84413, 84423, 84503, 84513, 84603, 84613, 84623, 84703, 84713, 84723, 84733, 84803, 84806, 84813, 849, 85002, 85003, 85012, 85013, 85023, 85032, 85033, 85042, 85043, 851, 852, 85303, 854, 85503, 85603, 85623, 857, 85803, 86003, 86203, 86303, 86403, 86503, 86803, 86933, 87003, 87103, 87202, 87203, 87213, 87223, 87233, 87303, 87403, 87412, 87413, 87422, 87423, 87433, 87443, 87453, 87613, 87703, 87713, 87723, 87733, 87743, 87803, 88003, 88006, 88013, 88023, 88033, 88043, 88103, 88113, 88123, 88133, 88143, 88303, 88323, 88333, 88403, 88503, 88513, 88523, 88533, 88543, 88553, 88583, 88903, 88913, 88943, 88953, 88963, 89003, 89013, 89023, 89103, 89203, 89303, 89333, 89403, 89413, 895, 89603, 89633, 89643, 897, 89803, 89813, 89903, 89913, 90003, 90203, 90403, 90413, 90423, 90433, 90443, 90503, 90513, 90523, 90533, 906, 90703, 90713, 90723, 90803, 90813, 90823, 90833, 90843, 90853, 90903, 91003, 91013, 91023, 91103, 91203, 91243, 91303, 91333, 91403, 91503, 91703, 91803, 91813, 91823, 91833, 91843, 91853, 91903, 92203, 92213, 92303, 92313, 92403, 92503, 92513, 92603, 92613, 92703, 92903, 93103, 93303, 93623, 93643, 93703, 93803, 93813, 93823, 93903, 93913, 93923, 940, 941, 942, 94303, 944, 945, 94603, 947, 948, 94903, 95003, 95013, 95023, 95033, 95043, 951, 952, 95303, 95393, 95403, 95603, 95613, 95803, 95813, 959, 965, 966, 967, 968, 969, 970, 971, 972, 973, 97403, 97413, 97603, 97613, 97623, 97633, 97643, 980, 982, 98303, 984, 98503, 986, 98703, 98803, 989, 99003, 99103, 993, 994, C00-C26, C30-C34, C37, C38- C86, C88, C90, C91-C97, D00-D09, Z85
	OHIP	OHIP DX :140-165, 170-175, 179- 208, 230-234
Stroke, including TIA	CIHI-DAD	ICD-10: I62, I630, I631, I632, I633, I634, I635, I638, I639, I64, H341, I600, I601, I602, I603, I604, I605, I606, I607, I609, I61, G450, G451, G452, G453, G458, G459, H340
Chronic liver disease	CIHI-DAD	ICD 10: B16, B17, B18, B19, I85, R17, R18, R160, R162, B942, Z225, E831, E830, K70, K713, K714, K715, K717, K721, K729, K73, K74, K753, K754, K758, K759, K76, K77
	OHIP	OHIP DX: 571, 573, 070<br>OHIP FEE: Z551, Z554
Coronary artery disease, with angina	CIHI-DAD	ICD-10: I20, I21, I22, I23, I24, I25, Z955, Z958, Z959, R931, T822<br>CCI: 1IJ26, 1IJ27, 1IJ54, 1IJ57, 1IJ50, 1IJ76<br>CCP: 4801, 4802, 4803, 4804, 4805, 481, 482, 483
	OHIP	OHIP DX: 410, 412, 413<br>OHIP FEE: R741, R742, R743, G298, E646, E651, E652, E654, E655, G262, Z434, Z448
Congestive heart failure	CIHI-DAD	ICD-10: I099, I420, I425, I426, I427, I428, I429, I43, I500, I501, I509, I255, J81<br>CCP: 4961, 4962, 4963, 4964<br>CCI: 1HP53, 1HP55, 1HZ53GRFR, 1HZ53LAFR, 1HZ53SYFR

© 2022 Muanda FT et al. *JAMA Network Open*.

Characteristic	Database	Codes
	OHIP	OHIP DX: 428<br>OHIP FEE: R701, R702, Z429
Epilepsy/seizure	CIHI-DAD	ICD-10: G40, G41, R5680, R5688
	OHIP	OHIP DX: 345, 780
Migraine	CIHI-DAD	ICD-10: G43
	OHIP	OHIP DX: 346
Rheumatoid Arthritis	CIHI-DAD	ICD10: M05, M06
	OHIP	OHIP Dx: 714
Dyslipidemia	CIHI-DAD	ICD-10: E78
	OHIP	OHIP DX: 272
Crohn disease	CIHI-DAD	ICD10: "K50"
	OHIP	OHIP DX: 555
Parkinson's disease	CIHI-DAD	ICD-9: 332<br>ICD-10: G20, F023
Peripheral vascular disease	CIHI-DAD	ICD 10: I700, I702, I708, I709, I731, I738, I739, K551<br>CCP: 5125, 5129, 5014, 5016, 5018, 5028, 5038, 5126, 5159<br>CCI: 1KA76, 1KA50, 1KE76, 1KG50, 1KG57, 1KG76MI, 1KG87, 1IA87LA, 1IB87LA, 1IC87LA, 1ID87, 1KA87LA, 1KE57
	OHIP	OHIP FEE: R787, R780, R797, R804, R809, R875, R815, R936, R783, R784, R785, E626, R814, R786, R937, R860, R861, R855, R856, R933, R934, R791, E672, R794, R813, R867, E649
Ulcerative colitis (UC)	CIHI-DAD	ICD10: K51
	OHIP	OHIP DX: 556
	OHIP	OHIP DX: 515
Osteortathritis	CIHI-DAD	ICD10: M15, M16, M17, M18, M19, M47
Gout	CIHI-DAD	ICD10: M10
	OHIP	OHIP dx: 274
Hypercalcemia	CIHI-DAD	ICD10: E835
Diabetes	ODB	Insulins, oral antihyperglycemic agents
Hypertension	ODB	Antihypertensive agents
Ventricular arrythmia	CIHI-DAD	ICD10: I4900, I472
Dementia	CIHI-DAD	ICD10: F065, F066, F068, F069, F09, F00, F01, F02, F03, F051, G30, G31, R54
	OHIP	OHIP DX CODES: "290", "331", "797"
	OMHRS (DSM-IV)	DSM-IV (OMHRS): "29040", "29041", "29042", "29043", "29120", "29282", "29410", "29411", "29480", "7809
Hyponatremia	CIHI-DAD	ICD10: E871

© 2022 Muanda FT et al. *JAMA Network Open*.

Characteristic	Database	Codes
Schizophrenia	CIHI-DAD	ICD-10: F060, F062, F105, F107, F115, F117, F125, F127, F135, F137, F145, F147, F155, F157, F165, F167, F175, F177, F185, F187, F195, F197, F200, F201, F202, F203, F204, "F205", "F206, F208, F209, F220, F228, F229, F230, F231, F232, F233, F238, F239, F24, F250, F251, F252, F258, F259, F28, F29
	OHIP	OHIP dx: 291, 292, 295, 297, 298<br>OHIP fee: Q021
	OMHRS (DSM-IV)	DSM-IV (OMHRS): 29130, 29150, 29211, 29212, 29381, 29382, 29510, 29520, 29530, 29540, 29560, 29570, 29590, 29710, 29730, 29880, 29890
Alcohol misuse	CIHI-DAD	ICD10: E24, E512, F10, G312, G621, G721, I426, K292, K70, K860, T510, X45, X65, Y15, Y573, Z502, Z714, Z721
	OHIP	OHIP dx: 303
hypotension	CIHI-DAD	ICD10: I95
Arrythmia	CIHI-DAD	ICD10: I48, I44, I45, I47, I4900, I4901, I491, I492, I493, I494, I498, I499, R000<br>R001<br>OHIP fee: G178, G179, G249, G261, G259 ,Z443,Z431 Z437
Urinary tract infection	CIHI-DAD	ICD10: N10, N11, N12, N136, N151, N159, N160, N300, N308, N309, N340, N390, N410, N411, N412, N413, N431, N45, T835
Community acquired pneumonia	CIHI-DAD	ICD10: J12, J13, J14, J15, J16, J17, J18, P23
Prosthetic joint infection	CIHI-DAD	ICD-10: T845
	OHIP	OHIP dx: 739
Other bacterial infections	CIHI-DAD	ICD-10: A49
	OHIP	OHIP dx: 786, 136, 040, 039
Gallstones /biliary stones	CIHI-DAD	ICD10: K80, K81, K82, K83, K87, K862, K863, K868, K869
	OHIP	OHIP DX: 574, 575, 576
Sepsis	CIHI-DAD	ICD10: A021, A392, A393, A394, A400, A401, A402, A408, A409, A410, A411, A412, A403, A414, A4159, A413, A4150, A4151, A4152, A4158, A4180" A4188, A427, A419
Chronic obstructive pulmonary disease	CIHI-DAD	ICD10: J41, J43, J44
Gastroesophageal reflux disease	CIHI-DAD	ICD10: K21
	OHIP	OHIP dx: 530, 531, 532, 533, 534, 535 , 536 , 537 , 538 , 539
Glaucoma	CIHI-DAD	ICD-10: H40<br>CCP: 0926
	OHIP	OHIP fee: "E123", "E133", "E214", "E983", "E984", "G819", "G820"

© 2022 Muanda FT et al. *JAMA Network Open*.

Characteristic	Database	Codes
Cataract	CIHI-DAD	ICD-10 H25, H26, H27, H28
	OHIP	OHIP fee: E214, E140, E141
Syncope	CIHI-DAD	ICD10: R55
Prostate cancer	CIHI-DAD	ICD10: C61, D075
	OHIP	OHIP DX: 185
Prostatitis	CIHI-DAD	ICD10: N410, N411, N412
	OHIP	OHIP DX: 601
Prostatic hyperplasia	CIHI-DAD	ICD10: N40
	OHIP	OHIP DX: 600
Macular degeneration	CIHI-DAD	ICD-10 H35
	OHIP	OHIP Fee: E154, E125, E126, E1
Obesity	CIHI-DAD	ICD10: E660, E661, E662, E668, E669
	OHIP	OHIP DX: 278
Inflammatory bowel disease	CIHI-DAD	ICD10: "K50", "K51"
Hypothyroidism	CIHI-DAD	ICD-10: E030, E031, E032, E033, E034, E035, E038, E039, E890
	OHIP	OHIP DX: 243, 244
Hypoglycemia	CIHI-DAD	ICD10: E15, E160, E161, E162, E1063, E1163, E1363, E1463
Pain	CIHI-DAD	ICD-10: F454, "M081", "M2550", "M2551", "M2555", "M2556", "M2557", "M432", "M433", M434", M435, M436", "M45", "M461", "M463", "M464", "M469", "M47", "M480", "M481", "M488", "M489", "M508", "M509", "M51", "M531", "M532", "M533", "M538", "M539", "M54", "M608", "M609", "M633", "M790", "M791", "M792", "M796", "M797", "M961", "G500,G530
Cirrhosis/liver damage	CIHI-DAD	ICD10: K702, K703, K704, K709, K740, K741, K742, K743, K744, K745, K746
	OHIP	OHIP DX: 571, 573
Chronic lung disease	CIHI-DAD	ICD10: I272, I278, I279, J40, J41, J42, J43, J44, J45, J47, J60, J61, J62, J63, J64, J65, J66, J67, J68, J701, J703, J704, J708, J709, J82, J84, J92, J941, J949, J953, J961, J969, J984, "J988", J989, J99
	OHIP	OHIP DX: 491, 492, 493, 494, 496, 501, 502, 515, 518, 519<br>OHIP FEE: J889, J689
Hypokalemia	CIHI-DAD	ICD10: E876
	OHIP	OHIP dx: 579
Urinary retention	CIHI-DAD	ICD10: R33
**Medication use (120 days before cohort entry)**		

© 2022 Muanda FT et al. *JAMA Network Open*.

Characteristic	Database	Codes
All medications	ODB	
**Healthcare Use (1 year prior to the cohort entry date)**		
GP/FP visits	OHIP<br>IPDB	Mainspeciality = "GP/FP" or "F.P./EMERGENCY MEDICINE"
Nephrologist visits	OHIP<br>IPDB	Mainspecialty = "NEPHROLOGY"
Number of any hospitalizations	CIHI-DAD	"ddate"
Number of any ER visits	NACRS	"regdate"
Number of serum creatinine tests	OLIS	OBSERVATIONCODE: 14682-9
TSH	OHIP	OHIP FEE: G016, L341
CT head	OHIP	OHIP FEE: X188, X400, X401, X402, X405, X408
CT abdomen	OHIP	OHIP FEE: X126, X409, X410
CT extremities	OHIP	OHIP FEE: X127, X412, X413
CT neck	OHIP	OHIP FEE: X124, X403, X404
CT pelvis	OHIP	OHIP FEE: X128, X415, X416
CT spine	OHIP	OHIP FEE: X231, X232, X233
CT thorax	OHIP	OHIP FEE: X125, X406, X407
Chest x-ray	OHIP	OHIP FEE: X090, X091, X092, X195
Echocardiography	CIHI-DAD	CCP: 0282<br>CCI: 3IP30
	OHIP	OHIP FEE: G560, G561, G562, G566, G567, G568, G570, G571, G572, G574, G575, G576, G577, G578, G581
Carotid ultrasound	CIHI-DAD	CCP: 0281<br>CCI: 3JE30, 3JG30
	OHIP	OHIP FEE: J201, J501, J190, J191, J490, J491, J492
Cardiac catheterization	CIHI-DAD	CCP: 4995, 4996, 4997, 4892, , 4893, 4894, 4895, 4896, 4897, 4898<br>CCI: 3IJ30GP, 3HZ30GP, 2HZ24GPKJ, 2HZ24GPKL, 2HZ24GPKM, 2HZ24GPXJ, 2HZ28GPPL, 2HZ71GP, 3IP10, 3IS10
	OHIP	OHIP FEE: G296, G297, G299, G300, G301, G304, G305, G306, G297, G509
Coronary angiogram	CIHI-DAD	CCP: 4892, 4893, 4894, 4895, 4896, 4897, 4898<br>CCI: 3IP10, 3IS10
	OHIP	OHIP FEE: G297, G509
Holter monitoring	CIHI-DAD	CCP: 0354<br>CCI: 2HZ24JAKH

© 2022 Muanda FT et al. *JAMA Network Open*.

Characteristic	Database	Codes
	OHIP	OHIP FEE: G311, G320, G647, G648, G649, G650, G651, G652, G653, G654, G655, G656, G657, G658, G659, G660, G661, G682, G683, G684, G685, G686, G687, G688, G689, G690, G692, G693
Cardiac stress test	CIHI-DAD	CCP: 0341, 0342, 0343, 0344, 0605<br>CCI: 2HZ08, 3IP70
	OHIP	OHIP FEE: G315, G174, G111, G112, G319, G582, G583, G584, J607, J608, J807, J808, J809, J866, J609, J666
Coronary revascularization	CIHI-DAD	CCP: 481, 482, 483, 480<br>CCI: 1IJ50, 1IJ26, IIJ27, 1IJ57, 1IJ76, 1IJ57GQ, 1IJ54GQAZ
	OHIP	OHIP FEE: R741, R742, R743, E651, E652, E654, E646, G298, Z434, G262
Electrocardiography	CIHI-DAD	CCI: 2HZ24JAKE
	OHIP	OHIP FEE: G310, G313
Colorectal cancer screening	OHIP	OHIP FEE: G004, L179, L181, Q043, Q152, X112, X113, Z535, Z536, Z555, Z580
Cervical cancer screening	OHIP	OHIP FEE: E430, G365, G394, L713, L812
Prostate-specific antigen test	OHIP	OHIP FEE: Q005, Q118, Q119, Q120, Q121, Q122, Q123, Q133
Mammography	OHIP	OHIP FEE: X172, X178, X184, X185, X201
Influenza vaccination	OHIP	OHIP FEE: G590, G591
Bone mineral density test	OHIP	OHIP FEE: J654, J688, J854, J888, X149, X152, X153, X155, Y654, Y688, Y854, Y888
Hearing test	OHIP	OHIP FEE: G153, G154, G440, G441, G442, G443, G448, G450, G451, G452, G525, G526, G529, G530, G533, G815, G816
Cystoscopy	OHIP	OHIP FEE: Z606, Z607, Z628, Z632, Z633, Z634
Pulmonary function test	OHIP	OHIP FEE: L354, L358
At-home physician service	OHIP	OHIP FEE: A901, B960, B961, B962, B963, B964, B966, B990, B992, B993, B994, B996, B997, B998
Sputum	OHIP	OHIP Fee: L629
Vaginal smear	OHIP	OHIP Fee: L625
Throat swab	OHIP	OHIP Fee: L640, L636
Urinalysis	OHIP	OHIP FEE: L253, L254, L255, L633, G009, G010
Serum creatinine value*	OLIS	OBSERVATIONCODE: 14682-9
Urine albumin-to-creatinine ratio*	OLIS	OBSERVATIONCODE: 14959-1, 30000-4,32294-1, XON10383-8 and XON12394-3

*Assessed in the 365-day period before the cohort entry date.

© 2022 Muanda FT et al. *JAMA Network Open*.

eTable 6. Justification for Using the CKD-EPI Equation to Estimate Patients' Glomerular Filtration Rate in This Study

The best equation to estimate GFR to guide drug-dosing adjustments in patients with chronic kidney disease remains controversial.[33,34] While the Cockcroft-Gault equation, expressed in mL/min, is a popular formula used to guide drug dosing, this equation requires information on body weight, which was not available in our data sources. However, the United States Kidney Disease Education program indicates that GFR equations that express results in mL/min per 1.73 m^2 or mL/min are appropriate to adjust drug doses in most adults. CKD-EPI eGFR <30 mL/min per 1.73 m^2 will usually also identify patients with a Cockcroft-Gault eGFR < 30mL/min.[35]

© 2022 Muanda FT et al. *JAMA Network Open*.

eTable 7. Median Dose of Fluoroquinolone Dispensed to Adults Aged 66 and Older With an Estimated Glomerular Filtration Rate <30 mL/min/ 1.73 m^2 in Ontario, Canada (2008 to 2020)

Fluoroquinolone	No. patients	Median dose, mg/day (range)
Ciprofloxacin	7614	500 (500 to 1000)
Levofloxacin	2492	500 (250 to 750)
Norfloxacin	1811	800 (400 to 800)

[a] Estimated with the chronic kidney disease–epidemiology equation.[35]

© 2022 Muanda FT et al. *JAMA Network Open*.

eTable 8. Operating Characteristics of Hospital Diagnosis Codes Used to Define the Primary and Secondary Outcomes

Outcome	Outcome component	ICD-10/CCI/OHIP/ORGD codes in this study	ICD-10 /CCI/OHIP /ORGD codes used in the validation study	Reference Standard	Operating Characteristics, % (95% CI)			Study
					Sensitivity	Specificity	Positive predictive value	
**Hospital visit with nervous system and/or psychiatric disorders** [a]	Delirium, not induced by alcohol and other psychoactive substance	<u>ICD-10 codes</u>: F050, F051, F058, F059, G934	<u>ICD-10 codes</u> F05, F10121, F10221, F10231, F10921, F11121, F11921, F12121, F12221, F12921, F13121, F13231, F13921, F13931, F14121, F14221, F14921, F15121, F15221, F15921, F16121, F16221, F16921, F18121, F18221, F18921, F19121, F19221, F19921 F19231, F19931 A812, E512, G0430, G0431, G0432, G0439, G92, G9340, G9341, G9349, I673, I674, I6783, J1081, J1181, P9160, P9161, P9163	Identification of delirium in a cohort of patients undergoing a cardiac surgery through interviews by geriatricians or trained research assistants	18 (10-30)	98 (93-100)	80 (52- 96)	Kim 2017[36]
	Disorientation unspecified	<u>ICD-10 code</u>: R410						
	Transient alteration of awareness	<u>ICD-10 codes</u>: R4180, R4188						
	Agitation and nervousness	<u>ICD-10 codes</u>: R451, R450						
	Somnolence	<u>ICD-10 code</u>: R400						

© 2022 Muanda FT et al. *JAMA Network Open*.

Outcome	Outcome component	ICD-10/CCI/OHIP/ORGD codes in this study	ICD-10 /CCI/OHIP /ORGD codes used in the validation study	Reference Standard	Operating Characteristics, % (95% CI)			Study
					Sensitivity	Specificity	Positive predictive value	
**Hospital visit with nervous system and/or psychiatric disorders** [a]	Dizziness and giddiness	ICD-10 code: R42						
	Peripheral neuropathy	ICD-10 codes: G603, G608, G611, G620						
**Hospital visit with hypoglycemia**		ICD-10 codes: E15, E160, E161, E162, E1063, E1163, E1363, E1463	ICD-10 codes: E15, E160, E161, E162, E1063, E1163, E1363, E1463	Positive predicted value: hypoglycemia in adults aged ≥65 on chart review defined as blood glucose <4 mmol/L or physician diagnosis<br><br>Sensitivity: hypoglycemia in adults aged ≥65 using healthcare databases with plasma glucose <4 mmol/L during hospital visit	13 (12-14)		94 (89-97)	Hodge 2017[37]

© 2022 Muanda FT et al. *JAMA Network Open*.

Outcome	Outcome component	ICD-10/CCI/OHIP/ORGD codes in this study	ICD-10 /CCI/OHIP /ORGD codes used in the validation study	Reference Standard	Operating Characteristics, % (95% CI)			Study
					Sensitivity	Specificity	Positive predictive value	
**Hospital visit with a collagen-associated event**[c]	Achilles' tendon rupture[b]	ICD 10 codes: S8600, S8608.<br>CCI codes: 1WT80<br>OHIP fee codes: R587, R589						
	Abdominal aortic aneurysm rupture	ICD10 codes: I713.<br>CCI codes: 1KA76, 1KA80<br>OHIP fee codes: R802, R817, R877, R875, E627	ICD-10 code: I713<br>CCI codes: 1KA76, 1KA80<br>OHIP fee codes: R802, R817, R875, E627<br>R877	Abdominal aortic aneurysm rupture in two academic tertiary hospitals on chart review by vascular surgeons			ICD-10 code<br>I713<br>83 (75,89)<br>CCI codes<br>1KA76<br>100 (74,100)<br>1KA80<br>100 (63,100)<br>OHIP fee codes<br>R802<br>100 (97,100)<br>R817<br>100 (97,100)<br>R875<br>98 (94,100)<br>E627<br>90 (83,96)<br>R877<br>100 (88,100)	Salata 2018[38]
**Hospital visit with sepsis**[d]		ICD-10 codes: A021, A392, A393, A394, A400, A401, A402, A408, A409, A410, A411, A412, A403, A414, A4159, A413, A4150, A4151, A4152, A4158, A4180, A4188, A427, "A419						
**Hospital visit with retinal detachments**		ICD-10 codes: H330, H331, H332, H333, H334, H335						

© 2022 Muanda FT et al. *JAMA Network Open*.

Outcome	Outcome component	ICD-10/CCI/OHIP/ORGD codes in this study	ICD-10 /CCI/OHIP /ORGD codes used in the validation study	Reference Standard	Operating Characteristics, % (95% CI)			Study
					Sensitivity	Specificity	Positive predictive value	
		OHIP fee codes: E152A, E148A, E142A, E936						
**Hospital visit with other tendinopathies**		ICD10 codes: M620, M621, M652, M661, M662, M6620, M663, M751, M752, M753, M754, M760, M761, M765, M766, M767, M768, M769, M775, M778, M779<br>OHIP fee codes: R589, R587, R578, E580, R585, E581, R557, E050, R559, E052, R586, R578, R585, R589, R593, R594, R530, R561, R584						
**Sudden cardiac death**		ORGD code: LCD_34, LCD_35, LCD_38, LCD_39, LCD_40, LCD_41, LCD_43						
**Death**					98	100		Jha P 1996[39]

Abbreviations: ICD-10, International Classification of Diseases, Tenth Revision; CCI code, Canadian Classification of Health Interventions code; OHIP codes, Ontario Health Insurance Program; Office of the registrar General Deaths (ORGD).

[a]The algorithm used to identify delirium and transient ischaemic attack has high specificity and high positive predicted value but low sensitivity. ICD-10 code algorithms to capture other components of encephalopathy were not validated. As such, we expected some outcome misclassification, but there is no reason to believe that misclassification occurred differentially between exposure groups.

[b]ICD-10 codes for falls were not validated. Therefore, outcome misclassification cannot be ruled out, but it is unlikely that this misclassification differ between comparison groups.

[c]ICD-10 codes and CCI codes for Achilles' tendon rupture have not been validated. We have also used OHIP fee codes to identify Achilles' tendon rupture. As such, we expect this outcome to be recorded accurately because Achilles' tendon rupture is linked to remuneration, and fee-for-service codes generally have high sensitivity and specificity.

[d] ICD -10 code algorithms to capture sepsis were not validated. As such, we expected some outcome misclassification, but there is no reason to believe that misclassification occurred differentially between exposure groups.

© 2022 Muanda FT et al. *JAMA Network Open*.

eTable 9. Variables Included in the Propensity Score Model

Category	Variables
**Demographics**	Age, sex, year of cohort entry, neighborhood income quintile, long-term residence, location, Local Health Integration Network, prescriber type
**Comorbidities**	Acute kidney injury, alcoholism, angina, bipolar disorder, chronic liver disease, chronic obstructive pulmonary disease, hyperkalemia, coronary artery disease (minus angina), dementia, diabetes, anemia, glaucoma, arrhythmia, congestive heart failure, hypertension, hypokalemia, hyponatremia, hypothyroidism, migraine, acute myocardial infarction, obesity, Parkinson disease, peripheral vascular disease, schizophrenia, hypoglycemia, seizure, ischemic stroke, hemorrhagic stroke, unipolar depression and/or anxiety disorder, rheumatoid arthritis, syncope, inflammatory bowel disease, cancer, prostatic hyperplasia, prostatitis, hypotension, ulcerative colitis, Crohn disease, acute urinary retention, macular degeneration, dyslipidemia, gastroesophageal reflux disease, osteoarthritis, transient ischemic stroke, Gallstones /biliary stones, gout, ventricular arrhythmia, community-acquired pneumonia, urinary tract infection, sepsis, modified Charlson comorbidity index
**Medications**	Alpha-adrenergic blocking agents, anti-arrhythmic, allopurinol, other antibiotics, anticoagulants, anticonvulsants, aspirin, antiplatelet agents, anticholinergics agent, bone calcium regulators, benzodiazepine, bisphosphonates,beta-agonists, calcium, chemotherapeutic drugs, cholinesterase inhibitors, glucocorticoid, nitrates, NSAIDs (excluding aspirin), opioids, overactive bladder medication, antipsychotics, proton pump inhibitors, 5 alpha reductases, selective serotonin reuptake inhibitors, statins, number of unique drug names, number of unique dins
**Health Care Use**	Emergency department visit, family physician visit, hospitalization
**Investigations**	Serum creatinine tests, TSH test, at home physician service, Bone mineral density test, cardiac catheterization, cardiac stress test, carotid ultrasound, chest-X ray, cataract surgery, cervical cancer screening, colorectal cancer screening, cholesterol test (total cholesterol, HDL), CT abdomen, CT extremities, CT head, CT neck, CT pelvis, CT spine, CT thorax, echocardiography, flu shot, cystoscopy, hearing test, mammography, prostate-specific antigen (PSA) test, Holter monitoring, parathyroid hormone testing, pulmonary function test, urinalysis, eGFR value, ACR

Abbreviations: ACR, urine albumin-to-creatinine ratio; ACE inhibitor, angiotensin-converting-enzyme inhibitor; CT, computed tomography; eGFR, estimated glomerular filtration rate.

© 2022 Muanda FT et al. *JAMA Network Open*.

eTable 10. Dose and Duration of Continuous Fluoroquinolone Dispensing in Older Adults With Advanced Chronic Kidney Disease Newly Prescribed a Fluoroquinolone in Ontario, Canada (2008-2020)

**Dose and duration of continuous fluoroquinolone dispensing**	**Higher dose**	**Lower dose**
	**n=5482 (46.0%)**	**n=6435 (54.0%)**
**Median daily dose, mg (IQR)**		
Ciprofloxacin	1000 (525 to 1000)	500 (500 to 500)
Levofloxacin	750 (523 to 750)	500 (250 to 500)
Norfloxacin	800 (457 to 800)	400 (400 to 400)
**Median duration,[a] days (IQR)**		
Ciprofloxacin	7 (7-10)	7 (6-10)
Levofloxacin	7 (5-8)	8 (7-10)
Norfloxacin	7 (6-9)	7 (5-10)

Abbreviations: IQR, interquartile range.

[a]Defined as consecutive prescription claims within a period equivalent to 150% of the days supplied for the previous prescription.

© 2022 Muanda FT et al. *JAMA Network Open*.

eTable 11. Baseline Characteristics of Older Adults With Advanced Chronic Kidney Disease Newly Prescribed a Fluoroquinolone in Ontario, Canada (2008-2020)[a]

		Unweighted data (N = 11,917)					Weighted data (N = 10,998)[b]				
		Higher dose (n = 5,482)		Lower dose (n = 6,435)		Standardized difference[c]	Higher dose (n = 5,482)		Lower dose (n = 5,516)		Standardized difference[c]
**Demographics**											
Age at cohort entry	Mean ± SD	82	8.0	83	8.2	23%	82	8.0	82	8.0	0%
	Median (IQR)	82	(76-88)	84	(77-90)		82	(76-88)	82	(75-88)	
	66-<70	493	9.0%	433	6.7%	9%	493	9.0%	512	9.3%	1%
	70-<75	706	12.9%	664	10.3%	8%	706	12.9%	753	13.7%	2%
	75-<80	944	17.2%	898	14.0%	9%	944	17.2%	908	16.5%	2%
	80-<85	1234	22.5%	1376	21.4%	3%	1234	22.5%	1255	22.7%	0%
	85-<90	1178	21.5%	1438	22.3%	2%	1178	21.5%	1095	19.9%	4%
	90+	927	16.9%	1626	25.3%	21%	927	16.9%	994	18.0%	3%
Sex	F	3287	60.0%	4151	64.5%	9%	3287	60.0%	3296	59.8%	0%
	M	2195	40.0%	2284	35.5%	9%	2195	40.0%	2219	40.2%	0%
Year of cohort entry	2008	395	7.2%	332	5.2%	8%	395	7.2%	412	7.5%	1%
	2009	664	12.1%	672	10.4%	5%	664	12.1%	690	12.5%	1%
	2010	718	13.1%	796	12.4%	2%	718	13.1%	713	12.9%	1%
	2011	626	11.4%	690	10.7%	2%	626	11.4%	641	11.6%	1%
	2012	553	10.1%	614	9.5%	2%	553	10.1%	560	10.1%	0%
	2013	481	8.8%	534	8.3%	2%	481	8.8%	488	8.8%	0%
	2014	434	7.9%	586	9.1%	4%	434	7.9%	422	7.7%	1%
	2015	373	6.8%	502	7.8%	4%	373	6.8%	368	6.7%	0%
	2016	319	5.8%	433	6.7%	4%	319	5.8%	316	5.7%	0%
	2017	256	4.7%	401	6.2%	7%	256	4.7%	258	4.7%	0%
	2018	291	5.3%	431	6.7%	6%	291	5.3%	286	5.2%	0%
	2019	308	5.6%	375	5.8%	1%	308	5.6%	302	5.5%	0%
	2020	64	1.2%	69	1.1%	1%	64	1.2%	59	1.1%	1%
Location	Urban	4846	88.4%	5724	89.0%	2%	4846	88.4%	4866	88.2%	1%
	Rural	636	11.6%	711	11.0%	2%	636	11.6%	650	11.8%	1%

© 2022 Muanda FT et al. *JAMA Network Open*.

		Unweighted data (N = 11,917)					Weighted data (N = 10,998)[b]				
		Higher dose (n = 5,482)		Lower dose (n = 6,435)		Standardized difference[c]	Higher dose (n = 5,482)		Lower dose (n = 5,516)		Standardized difference[c]
Residence	Long-term care	697	12.7%	1450	22.5%	26%	697	12.7%	686	12.4%	1%
LHIN	1	377	6.9%	366	6%	5%	377	6.9%	368	6.7%	1%
	2	544	9.9%	566	9%	4%	544	9.9%	528	9.6%	1%
	3	292	5.3%	466	7%	8%	292	5.3%	296	5.4%	0%
	4	758	13.8%	1144	18%	11%	758	13.8%	754	13.7%	0%
	5	243	4.4%	251	4%	3%	243	4.4%	244	4.4%	0%
	6	311	5.7%	332	5%	2%	311	5.7%	316	5.7%	0%
	7	349	6.4%	340	5%	5%	349	6.4%	353	6.4%	0%
	8	671	12.2%	633	10%	8%	671	12.2%	710	12.9%	2%
	9	652	11.9%	636	10%	6%	652	11.9%	668	12.1%	1%
	10	221	4.0%	290	5%	2%	221	4.0%	238	4.3%	2%
	11	510	9.3%	729	11%	7%	510	9.3%	500	9.1%	1%
	12	194	3.5%	210	3%	1%	194	3.5%	197	3.6%	1%
	13	265	4.8%	337	5%	2%	265	4.8%	258	4.7%	0%
	14	95	1.7%	135	2%	3%	95	1.7%	88	1.6%	1%
Socio-economic status[d]	1	1244	22.7%	1563	24%	4%	1244	22.7%	1265	22.9%	0%
	2	1224	22.3%	1434	22%	0%	1224	22.3%	1225	22.2%	0%
	3	1147	20.9%	1274	20%	3%	1147	20.9%	1164	21.1%	0%
	4	1001	18.3%	1138	18%	2%	1001	18.3%	994	18.0%	1%
	5	866	15.8%	1026	16%	0%	866	15.8%	869	15.7%	0%
**Prescriber information**											
General practitioner		4234	77.2%	5120	79.6%	6%	4234	77.2%	4241	76.9%	1%
Internal medicine		42	0.8%	94	1.5%	7%	42	0.8%	44	0.8%	0%
Nephrology		73	1.3%	293	4.6%	20%	73	1.3%	73	1.3%	0%
Urologist		402	7.3%	177	2.8%	21%	402	7.3%	427	7.7%	2%
Other		343	6.3%	263	4.1%	10%	343	6.3%	346	6.3%	0%

© 2022 Muanda FT et al. *JAMA Network Open*.

	Unweighted data (N = 11,917)					Weighted data (N = 10,998)[b]				
	Higher dose (n = 5,482)		Lower dose (n = 6,435)		Standardized difference[c]	Higher dose (n = 5,482)		Lower dose (n = 5,516)		Standardized difference[c]
Missing	388	7.1%	488	7.6%	2%	388	7.1%	385	7.0%	0%
**Comorbidities[e]**										
Acute kidney injury	1130	20.6%	1492	23.2%	6%	1130	20.6%	1151	20.9%	1%
Alcoholism	71	1.3%	77	1.2%	1%	71	1.3%	70	1.3%	0%
Angina	1396	25.5%	1601	24.9%	1%	1396	25.5%	1425	25.8%	1%
Atrial fibrillation/flutter	754	13.8%	1030	16.0%	6%	754	13.8%	761	13.8%	0%
bipolar disorder	136	2.5%	158	2.5%	0%	136	2.5%	128	2.3%	1%
Chronic liver disease	233	4.3%	233	3.6%	4%	233	4.3%	234	4.2%	0%
Anemia	2170	39.6%	2652	41.2%	3%	2170	39.6%	2217	40.2%	1%
Coronary artery disease (minus angina)	2264	41.3%	2725	42.3%	2%	2264	41.3%	2306	41.8%	1%
Congestive heart failure	1885	34.4%	2511	39.0%	10%	1885	34.4%	1903	34.5%	0%
cirrhosis	142	2.6%	154	2.4%	1%	142	2.6%	152	2.8%	1%
Chronic obstructive pulmonary disease	1577	28.8%	2102	32.7%	8%	1577	28.8%	1606	29.1%	1%
Dementia	1344	24.5%	2158	33.5%	20%	1344	24.5%	1350	24.5%	0%
Dyslipidemia	1225	22.3%	1357	21.1%	3%	1225	22.3%	1228	22.3%	0%
Glaucoma	400	7.3%	479	7.4%	0%	400	7.3%	400	7.3%	0%
Hypertension	5036	91.9%	5944	92.4%	2%	5036	91.9%	5096	92.4%	2%
Hypokalemia	209	3.8%	261	4.1%	2%	209	3.8%	213	3.9%	1%
Hyponatremia	148	2.7%	216	3.4%	4%	148	2.7%	147	2.7%	0%
Hypothyroidism	623	11.4%	751	11.7%	1%	623	11.4%	617	11.2%	1%
Hypoglycemia	146	2.7%	207	3.2%	3%	146	2.7%	154	2.8%	1%
Thyrotoxicosis	13	0.2%	21	0.3%	2%	13	0.2%	15	0.3%	2%
Migraine	130	2.4%	178	2.8%	3%	130	2.4%	126	2.3%	1%
Acute myocardial infarction	446	8.1%	574	8.9%	3%	446	8.1%	458	8.3%	1%
Obesity	305	5.6%	304	4.7%	4%	305	5.6%	326	5.9%	1%
Parkinson disease	131	2.4%	170	2.6%	1%	131	2.4%	125	2.3%	1%

© 2022 Muanda FT et al. *JAMA Network Open*.

	Unweighted data (N = 11,917)					Weighted data (N = 10,998)[b]				
	Higher dose (n = 5,482)		Lower dose (n = 6,435)		Standardized difference[c]	Higher dose (n = 5,482)		Lower dose (n = 5,516)		Standardized difference[c]
Peripheral vascular disease	189	3.4%	228	3.5%	1%	189	3.4%	185	3.4%	0%
Schizophrenia	218	4.0%	348	5.4%	7%	218	4.0%	215	3.9%	1%
Seizure	36	0.7%	56	0.9%	2%	36	0.7%	37	0.7%	0%
Hemorrhagic stroke	13	0.2%	20	0.3%	2%	13	0.2%	13	0.2%	0%
Ischemic stroke	195	3.6%	256	4.0%	2%	195	3.6%	194	3.5%	1%
Depression	532	9.7%	679	10.6%	3%	532	9.7%	544	9.9%	1%
Ventricular arrhythmia	40	0.7%	41	0.6%	1%	40	0.7%	36	0.7%	0%
Rheumatoid arthritis	332	6.1%	371	5.8%	1%	332	6.1%	340	6.2%	0%
Syncope	134	2.4%	217	3.4%	6%	134	2.4%	141	2.6%	1%
Inflammatory bowel disease	47	0.9%	43	0.7%	2%	47	0.9%	44	0.8%	1%
Major Cancer[f]	2337	42.6%	2597	40.4%	4%	2337	42.6%	2379	43.1%	1%
Prostatic hyperplasia	931	17.0%	770	12.0%	14%	931	17.0%	968	17.5%	1%
Prostatitis	179	3.3%	162	2.5%	5%	179	3.3%	184	3.3%	0%
Hypotension	178	3.2%	252	3.9%	4%	178	3.2%	180	3.3%	1%
Community acquired pneumonia	472	8.6%	758	11.8%	11%	472	8.6%	470	8.5%	0%
Coeliac disease	17	0.3%	13	0.2%	3%	17	0.3%	13	0.2%	2%
Ulcerative colitis	99	1.8%	75	1.2%	2%	99	1.8%	95	1.7%	1%
Crohn's disease	63	1.1%	60	0.9%	5%	63	1.1%	65	1.2%	1%
Acute urinary retention	302	5.5%	313	4.9%	2%	302	5.5%	316	5.7%	1%
Gallstones/biliary stones	296	5.4%	342	5.3%	3%	296	5.4%	299	5.4%	0%
Macula degeneration	384	7.0%	461	7.2%	0%	384	7.0%	388	7.0%	0%
Gastroesophageal reflux disease	1377	25.1%	1556	24.2%	1%	1377	25.1%	1414	25.6%	1%
Arrythmia	1016	18.5%	1353	21.0%	2%	1016	18.5%	1028	18.6%	0%
Osteoarthritis	387	7.1%	420	6.5%	6%	387	7.1%	399	7.2%	0%
Hyperkaliema	240	4.4%	327	5.1%	2%	240	4.4%	243	4.4%	0%
Prostate cancer	356	6.5%	325	5.1%	3%	356	6.5%	372	6.7%	1%

© 2022 Muanda FT et al. *JAMA Network Open*.

		Unweighted data (N = 11,917)					Weighted data (N = 10,998)[b]				
		Higher dose (n = 5,482)		Lower dose (n = 6,435)		Standardized difference[c]	Higher dose (n = 5,482)		Lower dose (n = 5,516)		Standardized difference[c]
Diabete		2107	38.4%	2353	36.6%	6%	2107	38.4%	2157	39.1%	1%
Urinary tract infection		998	18.2%	1266	19.7%	4%	998	18.2%	1030	18.7%	1%
Sepsis		188	3.4%	183	2.8%	3%	188	3.4%	196	3.6%	1%
Transient ischemic stroke		61	1.1%	112	1.7%	5%	61	1.1%	58	1.0%	1%
Gout		881	16.1%	969	15.1%	3%	881	16.1%	898	16.3%	1%
Other bacterial infections		2117	38.6%	2618	40.7%	4%	2117	38.6%	2145	38.9%	1%
Prosthetic joint infection		766	14.0%	854	13.3%	2%	766	14.0%	764	13.9%	0%
Prior fluoroquinolone associated adverse events		1517	27.7%	2043	31.7	9%	1517	27.7%	1622	29.4%	4%
Modified Charlson comorbidity index[g]	Mean ± SD	3.2	1.9	3.4	1.9	6%	3.2	1.9	3.2	1.8	1%
	Median (IQR)	2	(2-4)	2	(2-4)		2	(2-4)	2	(2-4)	
**Medication use (120-day look back)[h]**											
Alpha adrenergic blocking agents		486	8.9%	550	8.5%	1%	486	8.9%	501	9.1%	1%
Anti-arrhythmic		374	6.8%	511	7.9%	4%	374	6.8%	379	6.9%	0%
Other Antibiotics		1097	20.0%	1228	19.1%	2%	1097	20.0%	1129	20.5%	1%
Ace inhibitor		1741	31.8%	1953	30.3%	3%	1741	31.8%	1761	31.9%	0%
Anticoagulants		784	14.3%	1025	15.9%	4%	784	14.3%	777	14.1%	1%
Anticonvulsants		125	2.3%	165	2.6%	2%	125	2.3%	127	2.3%	0%
Angiotensin II receptor blockers		1436	26.2%	1579	24.5%	4%	1436	26.2%	1423	25.8%	1%
Aromatase inhibitors		34	0.6%	38	0.6%	0%	34	0.6%	35	0.6%	0%
Aspirin		172	3.1%	184	2.9%	1%	172	3.1%	180	3.3%	1%
Antiplatelet agents		643	11.7%	821	12.8%	3%	643	11.7%	664	12.0%	1%
Anticholinergic agents		480	8.8%	737	11.5%	9%	480	8.8%	485	8.8%	0%
Beta blockers		2564	46.8%	3058	47.5%	1%	2564	46.8%	2572	46.6%	0%
Bone calcium regulators		54	1.0%	120	1.9%	8%	54	1.0%	52	0.9%	1%
Benzodiazepine		953	17.4%	1125	17.5%	0%	953	17.4%	950	17.2%	1%

© 2022 Muanda FT et al. *JAMA Network Open*.

		Unweighted data (N = 11,917)					Weighted data (N = 10,998)[b]				
		Higher dose (n = 5,482)		Lower dose (n = 6,435)		Standardized difference[c]	Higher dose (n = 5,482)		Lower dose (n = 5,516)		Standardized difference[c]
Bisphosphonates		652	11.9%	766	11.9%	0%	652	11.9%	660	12.0%	0%
Beta agonist		521	9.5%	930	14.5%	15%	521	9.5%	525	9.5%	0%
Calcium		68	1.2%	85	1.3%	1%	68	1.2%	70	1.3%	1%
Calcium channel blocker		2558	46.7%	3070	47.7%	2%	2558	46.7%	2658	48.2%	3%
Chemotherapeutic drugs		84	1.5%	104	1.6%	1%	84	1.5%	88	1.6%	1%
Cholinesterase inhibitors		387	7.1%	551	8.6%	6%	387	7.1%	393	7.1%	0%
Glucocorticoid[i]		1621	29.6%	1912	29.7%	0%	1621	29.6%	1651	29.9%	1%
Loop diuretics		2389	43.6%	3185	49.5%	12%	2389	43.6%	2443	44.3%	1%
Nitrates		764	13.9%	1093	17.0%	9%	764	13.9%	782	14.2%	1%
NSAIDS (excluding ASA)		406	7.4%	351	5.5%	8%	406	7.4%	412	7.5%	0%
Opiods		1186	21.6%	1453	22.6%	2%	1186	21.6%	1214	22.0%	1%
Over-active bladder medication		247	4.5%	263	4.1%	2%	247	4.5%	261	4.7%	1%
Insulin		1086	19.8%	1322	20.5%	2%	1086	19.8%	1168	21.2%	3%
Anti-psychotics		425	7.8%	607	9.4%	6%	425	7.8%	432	7.8%	0%
Proton pump inhibitors		2372	43.3%	3018	46.9%	7%	2372	43.3%	2380	43.2%	0%
**Oral prednisone**		296	5.4%	401	6.2%	3%	296	5.4%	342	6.2%	3%
5 alpha reductases		239	4.4%	276	4.3%	0%	239	4.4%	249	4.5%	0%
Selective serotonin reuptake inhibitors		710	13.0%	1085	16.9%	11%	710	13.0%	694	12.6%	1%
Statins		3266	59.6%	3646	56.7%	6%	3266	59.6%	3330	60.4%	2%
Thiazide diuretics		913	16.7%	945	14.7%	5%	913	16.7%	915	16.6%	0%
Allopurinol		1035	18.9%	1079	16.8%	5%	1035	18.9%	970	17.6%	3%
Oral antidiabetics		1397	25.5%	1465	22.8%	6%	1397	25.5%	1360	24.6%	2%
Number of unique drug names	Mean ± SD	9.8	4.6	10.3	4.7	11%	9.8	4.6	9.8	4.3	2%
	Median (IQR)	9	(7-12)	10	(7-13)		9	(7-12)	9	(7-13)	
	Mean ± SD	10.7	5.5	11.4	5.7	13%	10.7	5.5	10.8	5.0	2%

© 2022 Muanda FT et al. *JAMA Network Open*.

		Unweighted data (N = 11,917)					Weighted data (N = 10,998)[b]				
		Higher dose (n = 5,482)		Lower dose (n = 6,435)		Standardized difference[c]	Higher dose (n = 5,482)		Lower dose (n = 5,516)		Standardized difference[c]
Number of unique dins	Median (IQR)	10	(7-14)	11	(7-15)		10	(7-14)	10	(7-14)	
**Healthcare use (365-day look back)**[j]											
GP/FP visits	Mean ± SD	12.7	12.7	13.8	13.3	9%	12.7	12.7	12.8	11.2	1%
	Median (IQR)	9	(5-15)				9	(5-15)	10	(5-15)	
Nephrology Visits	Mean ± SD	1.4	2.5	1.4	2.7	2%	1.4	2.5	1.5	2.5	4%
	Median (IQR)	0	(0-2)	0	(0-2)		0	(0-2)	0	(0-2)	
Number of hospitalizations	Mean ± SD	0.36	0.8	0.4	0.87	5%	0.36	0.8	0.36	0.77	0%
	Median (IQR)	0	(0-0)	0	(0-0)		0	(0-0)	0	(0-0)	
Number of emergency departments visits	Mean ± SD	0.96	1.57	0.99	1.6	2%	0.96	1.57	0.98	1.55	1%
	Median (IQR)	0	(0-1)	0	(0-1)		0	(0-1)	0	(0-1)	
Number of serum creatinine tests	Mean ± SD	4.1	3.8	4.4	3.9	7%	4.1	3.8	4.1	3.3	1%
	Median (IQR)	3	(2-5)	3	(2-6)		3	(2-5)	3	(2-5)	
TSH test		3787	69.1%	4428	68.8%	1%	3787	69.1%	3810	69.1%	0%
At home physician service		457	8.3%	640	9.9%	6%	457	8.3%	453	8.2%	0%
Bone mineral density test		295	5.4%	311	4.8%	3%	295	5.4%	299	5.4%	0%
Cardiac catheterization		77	1.4%	74	1.1%	3%	77	1.4%	78	1.4%	0%
Cardiac stress test		563	10.3%	628	9.8%	2%	563	10.3%	561	10.2%	0%
Carotid ultrasound		312	5.7%	349	5.4%	1%	312	5.7%	318	5.8%	0%
Chest-X ray		2458	44.8%	3293	51.2%	13%	2458	44.8%	2455	44.5%	1%
Cataract surgery		264	4.8%	273	4.2%	3%	264	4.8%	259	4.7%	0%
Cervical cancer screening		92	1.7%	88	1.4%	2%	92	1.7%	84	1.5%	2%

© 2022 Muanda FT et al. *JAMA Network Open*.

		Unweighted data (N = 11,917)					Weighted data (N = 10,998)[b]				
		Higher dose (n = 5,482)		Lower dose (n = 6,435)		Standardized difference[c]	Higher dose (n = 5,482)		Lower dose (n = 5,516)		Standardized difference[c]
Colorectal cancer screening		702	12.8%	672	10.4%	8%	702	12.8%	689	12.5%	1%
Cholesterol test (total cholesterol, HDL)		3503	63.9%	3894	60.5%	7%	3503	63.9%	3566	64.7%	2%
CT abdomen		715	13.0%	711	11.0%	6%	715	13.0%	733	13.3%	1%
CT extremities		56	1.0%	49	0.8%	2%	56	1.0%	60	1.1%	1%
CT head		662	12.1%	883	13.7%	5%	662	12.1%	666	12.1%	0%
CT neck		46	0.8%	42	0.7%	1%	46	0.8%	50	0.9%	1%
CT pelvis		685	12.5%	686	10.7%	6%	685	12.5%	699	12.7%	1%
CT spine		109	2.0%	127	2.0%	0%	109	2.0%	114	2.1%	1%
CT thorax		311	5.7%	379	5.9%	1%	311	5.7%	314	5.7%	0%
Echocardiography		1401	25.6%	1625	25.3%	1%	1401	25.6%	1404	25.5%	0%
Flu shot		2660	48.5%	2838	44.1%	9%	2660	48.5%	2717	49.3%	2%
Cytoscopy		429	7.8%	374	5.8%	8%	429	7.8%	454	8.2%	1%
Hearing test		234	4.3%	244	3.8%	3%	234	4.3%	231	4.2%	0%
Mammography		272	5.0%	262	4.1%	4%	272	5.0%	259	4.7%	1%
Prostate specific antigen (PSA) test		62	1.1%	51	0.8%	3%	62	1.1%	55	1.0%	1%
Holter monitoring		376	6.9%	466	7.2%	1%	376	6.9%	374	6.8%	0%
Parathytoid hormone testing		1491	27.2%	1779	27.6%	1%	1491	27.2%	1512	27.4%	0%
Pulmonary function test		433	7.9%	585	9.1%	4%	433	7.9%	433	7.9%	0%
Vaginal smear		38	0.7%	50	0.8%	1%	38	0.7%	34	0.6%	1%
Throat swab		64	1.2%	73	1.1%	1%	64	1.2%	54	1.0%	2%
Sputum		18	0.3%	34	0.5%	3%	18	0.3%	23	0.4%	2%
Urine culture		3833	69.9%	4189	65.1%	10%	3833	69.9%	3896	70.6%	2%
**Healthcare use (7-day look back)** [k]											

© 2022 Muanda FT et al. *JAMA Network Open*.

		Unweighted data (N = 11,917)					Weighted data (N = 10,998)[b]				
		Higher dose (n = 5,482)		Lower dose (n = 6,435)		Standardized difference[c]	Higher dose (n = 5,482)		Lower dose (n = 5,516)		Standardized difference[c]
CT abdomen		29	0.5%	38	0.7%	1%	29	0.5%	38	0.7%	3%
Chest-X ray		195	3.6%	318	5.8%	15%	195	3.6%	318	5.8%	10%
Urine culture		981	17.9%	936	17.0%	3%	981	17.9%	936	17.0%	2%
**Laboratory measurements**											
eGFR[l]	Mean ± SD	23.9	5.0	23.3	5.2	12%	23.9	5.0	23.9	4.6	0%
Most recent eGFR value	Median (IQR)	25	(21-28)	24	(20-28)		25	(21-28)	25	(21-28)	
Urine ACR available		2366	43.2%	2604	40.5%	5%	2366	43.2%	2412	43.7%	1%
Baseline ACR categories, μg/mg	Missing	3116	56.8%	3831	59.5%	5%	3116	56.8%	3104	56.3%	1%
	<30	683	12.5%	682	10.6%	6%	683	12.5%	655	11.9%	2%
	30-300	947	17.3%	1033	16.1%	3%	947	17.3%	961	17.4%	0%
	>300	736	13.4%	889	13.8%	1%	736	13.4%	796	14.4%	3%

Abbreviations: eGFR, estimated glomerular filtration rate; IQR, interquartile range; LHIN, Local Health Integration Network; ACR, urine albumin-to-creatinine ratio.

[a] Unless otherwise specified in the footnotes, baseline characteristics were assessed on the date the patient filled a fluoroquinolone prescription—the cohort entry date.

[b] Weighted using inverse probability of exposure weighting based on propensity scores. The propensity score was estimated using multivariable logistic regression with 121 covariates chosen *a priori* (defined in eTable 9 in the Supplement). Patients in the reference group were weighted as [propensity score/ (1 - propensity score)].[40-42] This method produces a weighted pseudo-sample of patients in the reference group with the same distribution of measured covariates as the exposure group.[40,41]

[c] The difference between the groups divided by the pooled SD; a value greater than 10% is interpreted as a meaningful difference.[43]

[d] Income was categorized into fifths of average neighborhood income on the cohort entry date.

[e] Baseline comorbidities were assessed in the 5-year period before the cohort entry date.

[f] Cancer includes the following types of cancer: skin, mouth (lip, tonsil, etc), throat, stomach, small/large intestine, liver, gall bladder, pancreas, breast, male/female reproductive organs, heart, lung, bone, urinary system (kidney, bladder, etc), endocrine glands, as well as leukemias and lymphomas

[g] Presence of kidney disease is a variable in the Charlson comorbidity index, which automatically results in all individuals receiving a minimum score of 2. Individuals with a Charlson comorbidity index of 0 were given a score of 2, and individuals with a score of 1 were given a score of 3.

© 2022 Muanda FT et al. *JAMA Network Open*.

[h] Medication use was examined in the 120-day period before the cohort entry date (the Ontario Drug Benefit program dispenses a maximum 100-day supply).

[I] Glucocorticoids included many medications regardless of their route of administration such as hydrocortisone acetate, dexamethasone, beclomethasone dipropionate, prednisone, hydrocortisone, flumetasone pivalate, clioquinol, betamethasone valerate, betamethasone, triamcinolone acetonide, triamcinolone diacetate, triamcinolone, flurandrenolide, betamethasone & dexamethasone sodium phosphate, cortisone acetate, dexamethasone tebutate, prednisolone, dexamethasone, corticotrophin, prednisolone acetate, fluocinolone acetonide, hydrocortisone sodium succinate, methylprednisolone sodium succinate, methylprednisolone acetate, methylprednisolone disodium phosphate, methylprednisolone, fluocinonide, betamethasone disodium phosphate, medrysone & polyvinyl alcohol, prednisolone acetate & sulfacetamide sodium, dexamethasone & neomycin sulfate & polymyxin b sulfate, clioquinol & flumetasone pivalate, clioquinol & hydrocortisone, 1,2-propanediol diacetate & acetic acid & benzethonium chloride & hydrocortisone, clioquinol & triamcinolone acetonide, flurandrenolide, fluocinolone acetonide, dexamethasone & neomycin sulfate, hydrocortisone & lidocaine hcl & neomycin sulfate, haemorroidal veinous plexus, prednisone & pheniramine maleate & inositol & phosphatidyl choline & vitamin a & vitamin d2& vitamin e, chloramphenicol & hydrocortisone acetate, haemorroidal veinous plexus, dexamethasone & framycetin sulfate & gramicidin, dibucaine hcl & esculin & framycetin sulfate & hydrocortisone, betamethasone valerate & neomycin sulfate, betamethasone valerate & gentamicin sulfate, prednisolone acetate & sulfacetamide sodium, ascorbic acid & chlorpheniramine maleate & prednisone acetate, neomycin sulfate & prednisolone acetate & sulfacetamide sodium, gramicidin & neomycin sulfate & triamcinolone acetonide, methylprednisolone, acetylsalicylic acid & methyltestosterone, methylprednisolone sulfate & neomycin sulfate, hydrocortisone acetate & neomycin sulfate, aluminum chlorohydrate & methylprednisolone acetate & neomycin sulfate & sulfur, gramicidin & neomycin sulfate & nystatin & triamcinolone acetonide, hydrocortisone acetate & zinc oxide, hydrocortisone acetate & pramoxine hcl & zinc sulfate, aluminum chlorohydrate & methylprednisolone acetate & sulfur, hydrocortisone acetate & zinc oxide, hydrocortisone acetate & pramoxine hcl & zinc sulfate, desonide, clobetasol propionate, beclomethasone dipropionate & clioquinol, bacitracin zinc & hydrocortisone & neomycin sulfate & polymyxin b sulfate, hydrocortisone & neomycin sulfate & polymyxin b sulfate, gramicidin & neomycin sulfate & nystatin & triamcinolone acetonide, fluorometholone & polyvinyl alcohol, aluminum chlorohydrate & methylprednisolone acetate & sulfur, fluorometholone, lidocaine hcl & methylprednisolone acetate, flumetasone pivalate & salicylic acid, fluorometholone, lidocaine hcl & methylprednisolone acetate, aclometasone dipropionate, allantoin & chloramphenicol & hydrocortisone, amcinonide, atropine sulfate & prednisolone acetate, bacitracin & hydrocortisone & neomycin sulfate & polymyxin b sulfate, benzalkonium & dexamethasone & tobramycin , benzocaine & hydrocortisone acetate & zinc sulfate, betamethasone & sulfacetamide sodium, betamethasone acetate & betamethasone sodium phosphate, betamethasone benzoate, betamethasone dipropionate, betamethasone dipropionate &calcipotriene, betamethasone dipropionate &clotrimazole, betamethasone dipropionate & gentamicin sulfate, betamethasone dipropionate & salicylic acid, betamethasone disodium phosphate, betamethasone valerate & salicylic acid, betamethasone valerate & gentamycin sulfate, betamethasone valerate & neomycin sulfate, budesonide, camphor & hydrocortisone & menthol, chlorbutol & dexamethasone &tobramycin, ciclesonide.

[j] Total number of healthcare visits/tests in the 12-month period before the cohort entry date.

[k] Total number of healthcare visits/tests in the 7-day period before the cohort entry date

[l] The most recent eGFR measurement in the 365 day period before the cohort entry date (including the cohort entry date); eGFR was calculated using the Chronic Kidney Disease (CKD)–Epidemiology (EPI) equation: $141 \times \min([\text{serum creatinine concentration in } \mu\text{mol/L}/88.4]/\kappa, 1)^{\alpha} \times \max([\text{serum creatinine concentration in } \mu\text{mol/L}/88.4]/\kappa, 1)^{-1.209} \times 0.993^{Age} \times 1.018$ [if female] $\times$ 1.159 [if African-American]; κ=0.7 if female and 0.9 if male; α=-0.329 if female and -0.411 if male; min=the minimum of serum creatinine concentration/κ or 1; max=the maximum of serum creatinine concentration/κ or 1. all patients were assumed not to be of African-Canadian race; African-Canadians represented less than 5% of the population of Ontario in 2006.

eTable 12. Post Hoc Survival Analysis in Older Adults With Advanced Chronic Kidney Disease Within 14 Days of Starting a New Prescription for a Higher- Vs Lower-Dose Fluoroquinolone: Risk of a Hospital Visit With Nervous System and/or Psychiatric Disorders, Hypoglycemia, or a Collagen-Associated Event

© 2022 Muanda FT et al. *JAMA Network Open*.

Fluoroquinolone dose		Unweighted			Weighted[a]			
		No. patients	No. events (%)	Event rate per 1000 person-years	No. patients	No. events (%)	Event rate Per 1000 person-years	Hazard ratio (95% CI)
**Exposure**	Higher-dose[b]	5482	68 (1.2)	327.8	5482	68 (1.2)	327.8	1.45 (1.01 to 2.09)[c]
**Referent**	Lower-dose[b]	6435	67 (1.0)	276.7	5516	47 (0.85)	225.0	

[a]Inverse probability of treatment weighting on the propensity score was used to balance comparison groups on indicators of baseline health.[40-42] The propensity score was estimated using multivariable logistic regression with 121 covariates chosen *a priori* (defined in eTable 9 in the Supplement). Patients in the reference group were weighted as [propensity score/ (1 - propensity score)].[40-42] This method produces a weighted pseudo-sample of patients in the reference group with the same distribution of measured covariates as the exposed group.[40,41]

[b] Higher-dose : ciprofloxacin 501 to 1000 mg/day, levofloxacin 501 to 750 mg/day, or norfloxacin 401 to 800 mg/day. Lower-dose fluoroquinolone: ciprofloxacin 500 mg/day, levofloxacin 250 to 500 mg/day, or norfloxacin 400 mg/day.

[c] We used a Cox proportional hazards regression (with 14-day follow-up censoring on death) to estimate the 14-day risk of a hospital admission or emergency department visit with nervous system and/or psychiatric disorders , hypoglycemia, or a collagen-associated event.

eTable 13. Risk of Heart Failure in Older Adults With Advanced Chronic Kidney Disease Within 14 Days of Starting a New Prescription for a Higher- vs Lower-Dose Fluoroquinolone[a]

© 2022 Muanda FT et al. *JAMA Network Open*.

	Unweighted		Weighted[b]			
	No. events (%)		No. events (%)			
	fluoroquinolone dose		fluoroquinolone dose			
Outcome	Higher dose (n = 5,482)	Lower dose (n = 6,435)	Higher dose (n = 5,482)	Lower dose (n = 5,516)	Risk difference, % (95% CI)	Risk ratio (95% CI)
Hospital admission with heart failure (main diagnosis)	22 (0.40)	27 (0.42)	22 (0.40)	21 (0.37)	0.03 (-0.20 to 0.26)	1.07 (0.59 to 1.95)

[a] Higher-dose : ciprofloxacin 501 to 1000 mg/day, levofloxacin 501 to 750 mg/day, or norfloxacin 401 to 800 mg/day. Lower-dose fluoroquinolone: ciprofloxacin 500 mg/day, levofloxacin 250 to 500 mg/day, or norfloxacin 400 mg/day.

[b]The propensity score was estimated using multivariable logistic regression with 121 covariates chosen *a priori* (defined in eTable 9 in the Supplement).[40-42] Patients in the reference group were weighted as [propensity score/(1 - propensity score)].[40-42] This method produces a weighted pseudo-sample of patients in the reference group with the same distribution of measured covariates as the exposed group.[40,41] Weighted risk ratios and 95% CIs were obtained using modified Poisson regression[44] and weighted risk differences and 95% CIs were obtained using a binomial regression model with an identity link function.

© 2022 Muanda FT et al. *JAMA Network Open*.

eTable 14. Risk of a Hospital Visit With Nervous System and/or Psychiatric Disorders, Hypoglycemia, or a Collagen-Associated Event in Older Adults With Advanced Chronic Kidney Disease Within 14 Days of Starting a New Prescription for a Higher- vs Lower-Dose Fluoroquinolone[a] Using Fine Stratification Weighting[b]

	**Unweighted**		**Weighted**			
	**No. events (%)**		**No. events (%)**			
	**fluoroquinolone dose**		**fluoroquinolone dose**			
**Outcome**	**Higher dose (n = 5,482)**	**Lower dose (n = 6,435)**	**Higher dose (n = 5,480)**	**Lower dose (n = 6,411)**	**Risk difference, % (95% CI)**	**Risk ratio (95% CI)**
Hospital visit with nervous system and/or psychiatric disorders , hypoglycemia, or a collagen-associated event	68 (1.2)	67 (1.0)	68 (1.2)	54 (0.84)	0.39 (0.02 to 0.77)	1.47 (1.02 to 2.11)

[a] Higher-dose : ciprofloxacin 501 to 1000 mg/day, levofloxacin 501 to 750 mg/day, or norfloxacin 401 to 800 mg/day. Lower-dose fluoroquinolone: ciprofloxacin 500 mg/day, levofloxacin 250 to 500 mg/day, or norfloxacin 400 mg/day.

[b] This weighting method does not use the propensity score directly to calculate weights; instead, propensity scores are used to create fine stratum after ranking only the exposed patients (ciprofloxacin 501 to 1000 mg/day, levofloxacin 501 to 750 mg/day, or norfloxacin 401 to 800 mg/day) based on the propensity score and assigning unexposed patients (ciprofloxacin 500 mg/day or levofloxacin 250 to 500 mg/day or norfloxacin 400mg/day) to these strata based on their propensity score. The weights for the exposed group are set to 1 and reference patients are re-weighted based on the number of exposed patients residing within their stratum, so that unexposed patients contribute proportionally to the relative number of total patients within a stratum. Patients in the unexposed group are weighted as ($N_{\text{exposed in PS stratum i}}/N_{\text{total exposed}}$) / ($N_{\text{unexposed in PS stratum i}}/N_{\text{total unexposed}}$). This weighting creates a pseudo-population in which confounder distribution concordance is achieved between the exposed and unexposed groups, to the extent that it is achieved within each stratum. As a result, extreme weights due to propensity scores that are very close to 0 or 1 are unlikely.[45,46] This method calculates a treatment effect estimate similar to the propensity score matching estimate (i.e., an average treatment effect among the treated population, ATT).[45,46]

.

© 2022 Muanda FT et al. *JAMA Network Open*.

eFigure 1. Flow Diagram of Cohort Build

Source population (N = 1,277,043)
Older adults in Ontario newly prescribed an oral fluoroquinolone as outpatients from January 1, 2008, to March 17, 2020

Patients excluded from the study (N = 1,265,126)

- Died on the fluoroquinolone dispensing date, or non-Ontario resident (data cleaning), n = 1282
- Age less than 66 years on the fluoroquinolone dispensing date, n = 55,229
- Prescription for any fluoroquinolone in the prior 180 days, n = 278,627
- Prescription of unusual dose of fluoroquinolone (ciprofloxacin <500 mg/day or >1000 mg/day; norfloxacin <400 mg/day or >800 mg/day; levofloxacin <250 mg/day or >750 mg/day), n = 21,860
- Prescription of more than one fluoroquinolone on index date, n = 158
- Kidney failure, n = 14,990
- Hospital discharge or emergency department visit in the 2-day period before the fluoroquinolone dispensing date, n = 229,930
- Patients without an outpatient baseline serum creatinine value in the year before the fluoroquinolone dispensing date, n =302,629
- Baseline eGFR $\geq$ 30 mL/min per $1.73m^2$, n = 360,421

Patients included in the study (N = 11,917)
Older adults with an eGFR < 30 mL/min per $1.73m^2$
newly prescribed an oral fluoroquinolone
Higher dose, **N = 5,482 (46.0%)**
Lower dose, **N = 6,435 (54.0%)**

© 2022 Muanda FT et al. *JAMA Network Open*.

eFigure 2. e-Value Analysis to Assess the Extent of Unmeasured Confounding That Would Be Required to Negate the Observed Results

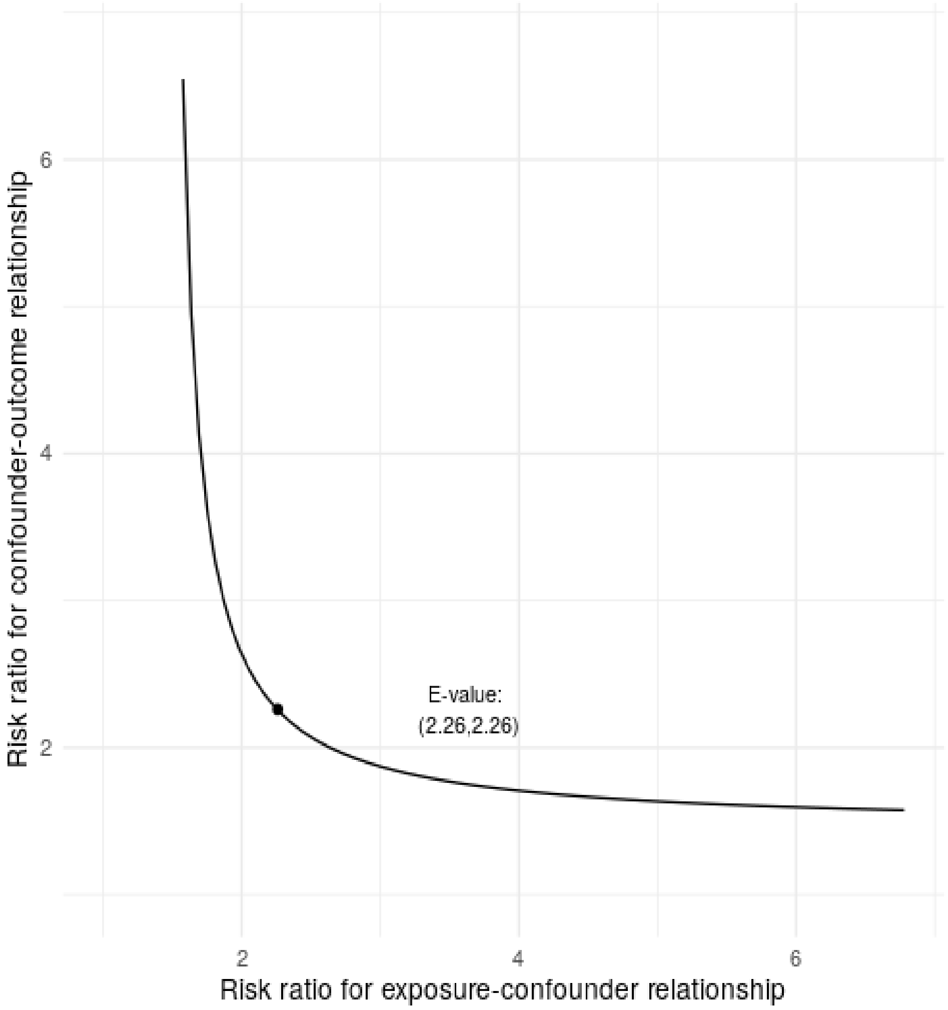

E-value for point estimate: 2.26 and for confidence interval: 1.11

© 2022 Muanda FT et al. *JAMA Network Open*.

eReferences

1. Ciprofloxacin: Drug information - UpToDate. https://www.uptodate.com/contents/ciprofloxacin-systemic-drug-information?search=ciprofloxacin%20adult&source=panel_search_result&selectedTitle=1~147&usage_type=panel&display_rank=1. Accessed October 19, 2021.

2. Ciprofloxacin (Product Monograph). http://auropharma.ca/products/monograph/Auro-Ciprofloxacin-PM.pdf. Accessed October 19, 2021.

3. Levofloxacin: Drug information - UpToDate. https://www.uptodate.com/contents/levofloxacin-systemic-druginformation?search=levofloxacin%20adult&source=panel_search_result&selectedTitle=1~146&usage_type=panel&display_rank=1 Accessed October 19, 2021

4. Levofloxacin (Product Monograph). https://www.sandoz.ca/sites/www.sandoz.ca/files/Levofloxacin_TAB_Monograph.pdf Accessed October 19, 2021.

5. Norfloxacin: Drug information - UpToDate https://www.uptodate.com/contents/norfloxacin-united-states-not-available-drug-information?search=norfloxacin%20adult&source=panel_search_result&selectedTitle=1~28&usage_type=panel&kp_tab=drug_general&display_rank=1. Accessed October 19, 2021

6. Norfloxacin (Product Monograph). https://pdf.hres.ca/dpd_pm/00043817.PDF. Accessed October 19, 2021.

7. Abdalla A, Ramly S, Boers P, Casserly LJCR. Ciprofloxacin-associated choreoathetosis in a haemodialysis patient. 2013;2013:bcr2013009293.

8. Stroud SG, Kandemir UJJcc. Acute Delirium Induced by Ciprofloxacin in a Patient With Chronic Kidney Disease: A Case Report. 2020;10(2):e0603.

9. Schwalm J-D, Lee CHJC. Acute hepatitis associated with oral levofloxacin therapy in a hemodialysis patient. 2003;168(7):847-8.

10. Abo-Salem E, Nugent K, Chance WJJotAGS. Antibiotic-induced cardiac arrhythmia in elderly patients. 2011;59(9):1747-9.

11. Kawtharani F, Masrouha KZ, Afeiche NJTJoF, Surgery A. Bilateral Achilles tendon ruptures associated with ciprofloxacin use in the setting of minimal change disease: case report and review of the literature. 2016;55(2):276-8.

12. Denysenko L, Nicolson SEJP. Cefoxitin and ciprofloxacin neurotoxicity and catatonia in a patient on hemodialysis. 2011;52(4):379-83.

13. Martin M, Boixeda R, Muñoz A, Felip ÀJEiymc. Ciprofloxacin as a cause of acute renal failure. 2020.

14. Sedlacek M, Suriawinata AA, Schoolwerth A, Remillard BDJNDT. Ciprofloxacin crystal nephropathy—a ‘new’cause of acute renal failure. 2006;21(8):2339-40.

15. . Reece RJ, Nicholls AJ. Ciprofloxacin-induced acute interstitial nephritis. Nephrology, dialysis, transplantation: official publication of the European Dialysis and Transplant Association - European Renal Association. 1996;11(2):393.

16. Striano P, Zara F, Coppola A, Ciampa C, Pezzella MJMd. Epileptic myoclonus as ciprofloxacin-associated adverse effect. 2007;22(11):1675-6.

© 2022 Muanda FT et al. *JAMA Network Open*.

17. Matoi A, Taguchi M, Nishi SJCCR. Fatal hypoglycemia with ciprofloxacin in a dialysis patient: A case report. 2021;9(4):1902.

18. Marti H, Stoller R, Frey FJBjor. Fluoroquinolones as a cause of tendon disorders in patients with renal failure/renal transplants. 1998;37(3):343-4.

19. Takeda S-i, Imai T, Chaki Y, Kusano EJC, nephrology e. Four consecutive cases of Achilles tendon disorders associated with levofloxacin treatment in hemodialysis patients. 2012;16(6):977.

20. Idrees N, Almeqdadi M, Balakrishnan VS, Jaber BLJHI. Hemodialysis for treatment of levofloxacin-induced neurotoxicity. 2019;23(2):E40-E5.

21. Gkoufa A, Sakellariou S, Katsoulas N, Georgakopoulou VE, Lazaris A, Cholongitas EJDT. Henoch-Schönlein purpura associated with ciprofloxacin. 2020:e14591.

22. Korzets A, Gafter U, Dicker D, Herman M, Ori YJNDT. Levofloxacin and rhabdomyolysis in a renal transplant patient. 2006;21(11):3304-5.

23. Kato A, Ishigaki S, Yasuda HJC, nephrology e. Levofloxacin-associated Achilles tendinitis in a patient with chronic kidney disease stage 5. 2011;15(2):318-9.

24. Nishikubo M, Kanamori M, Nishioka HJA. Levofloxacin-Associated Neurotoxicity in a Patient with a High Concentration of Levofloxacin in the Blood and Cerebrospinal Fluid. 2019;8(2):78.

25. Patil SS, Patil SM, Campbell R, Singh M, Plotkin MJCRiM. Levofloxacin-Induced Acute Hyperpigmentation Changes in a Chronic Kidney Disease Patient. 2020;2020.

26. Majda A, Rostoff P, Nessler J, Gajos GJCD. Levofloxacin-induced life-threatening hypoglycemia in a type 2 diabetic patient with ST-segment elevation myocardial infarction and community-acquired pneumonia. 2020;9(2):141-3.

27. . Kelesidis T, Canseco EJTAjom. Quinolone-induced hypoglycemia: a life-threatening but potentially reversible side effect. 2010;123(2):e5.

28. Tsai L-H, Weng Y-M, Lin C-C, Kuo C-W, Chen J-CJTAjoem. Risk screening for long QT prior to prescribing levofloxacin. 2014;32(9):1153. e1-. e3.

29. Kushner JM, Peckman HJ, Snyder CRJAoP. Seizures associated with fluoroquinolones. 2001;35(10):1194-8.

30. . Parra-Riffo H, Lemus-Penaloza JJN. Severe levofloxacin-induced hypoglycaemia: a case report and literature review. 2012;32(4):546-7.

31. . Proietti R, Rognoni A, Maccio S, Corrado L, Rognoni GJAoL-TC. Torsades de pointes after fluoroquinolone therapy in an elderly patient with comorbidities.19:35-9.

32. Assimon MM, Pun PH, Wang LC, Al-Khatib SM, Brookhart MA, Weber DJ, Winkelmayer WC, Flythe JE. Analysis of Respiratory Fluoroquinolones and the Risk of Sudden Cardiac Death Among Patients Receiving Hemodialysis. JAMA Cardiol. 2021 Oct 20. doi: 10.1001/jamacardio.2021.4234. Epub ahead of print. PMID: 34668928.

33. Matzke GR, Aronoff GR, Atkinson AJ, Jr., et al. Drug dosing consideration in patients with acute and chronic kidney disease-a clinical update from Kidney Disease: Improving Global Outcomes (KDIGO): Kidney Int. 2011 Dec;80(11):1122-37. doi: 10.1038/ki.2011.322. Epub 2011 September 14.

34. .NIDDK: Estimating glomerular filtration rate. Available at: https://www.niddk.nih.gov/health-information/communicationprograms/nkdep/laboratory-evaluation/glomerular-filtration-rate/estimating. Accessed April 15, 2021.

© 2022 Muanda FT et al. *JAMA Network Open*.

35. Levey AS, Stevens LA. Estimating GFR using the CKD Epidemiology Collaboration (CKD-EPI) creatinine equation: more accurate GFR estimates, lower CKD prevalence estimates, and better risk predictions. Am J Kidney Dis. Apr 2010;55(4):622-627.

36. Kim DH, Lee J, Kim CA, et al. Evaluation of algorithms to identify delirium in administrative claims and drug utilization database. Pharmacoepidemiology and drug safety. 2017;26(8):945-953 S29.

37. Hodge MC, Dixon S, Garg AX, Clemens KK. Validation of an International Statistical Classification of Diseases and Related Health Problems 10th Revision Coding Algorithm for Hospital Encounters with Hypoglycemia. Can J Diabetes. 2017;41(3):322-328.

38. Salata K, Hussain MA, De Mestral C, Greco E, Mamdani M, Tu JV, Forbes TL, Verma S, Al-Omran M. Validation of abdominal aortic aneurysm repair codes in Ontario administrative data. Clin Invest Med. 2018 Sep 30;41(3):E148-E155. doi: 10.25011/cim.v41i3.30858. PMID: 30315751.

39. Jha P, Deboer D, Sykora K, Naylor CD: Characteristics and mortality outcomes of thrombolysis trial participants and nonparticipants: A population-based comparison. J Am Coll Cardiol 27: 1335– 1342, 1996.

40. Sato T, Matsuyama Y. Marginal structural models as a tool for standardization. Epidemiology (Cambridge, Mass). 2003;14(6):680-686.

41. Brookhart MA, Wyss R, Layton JB, Sturmer T. Propensity score methods for confounding control in nonexperimental research. Circ Cardiovasc Qual Outcomes. 2013;6(5):604-61

42. Austin PC. An Introduction to Propensity Score Methods for Reducing the Effects of Confounding in Observational Studies. Multivariate Behav Res. 2011;46(3):399-424.

43. Austin PC, Grootendorst P, Anderson GM. A comparison of the ability of different propensity score models to balance measured variables between treated and untreated subjects: a Monte Carlo study. Stat Med. 2007;26(4):734-753.

44. Zou G. A modified poisson regression approach to prospective studies with binary data. *American journal of epidemiology.* 2004;159(7):702-706.

45. Desai RJ, Franklin JM. Alternative approaches for confounding adjustment in observational studies using weighting based on the propensity score: a primer for practitioners. BMJ (Clinical research ed). 2019;367: l5657.

46. Desai RJ, Rothman KJ, Bateman BT, Hernandez-Diaz S, Huybrechts KF. A propensity-score-based fine stratification approach for confounding adjustment when exposure is infrequent. Epidemiology 2017; 28:249-57. doi:10.1097/ EDE.0000000000000595

© 2022 Muanda FT et al. *JAMA Network Open*.
